# circCUL2 induces an inflammatory CAF phenotype in pancreatic ductal adenocarcinoma via the activation of the MyD88-dependent NF-κB signaling pathway

**DOI:** 10.1186/s13046-021-02237-6

**Published:** 2022-02-21

**Authors:** Shangyou Zheng, Chonghui Hu, Hongcao Lin, Guolin Li, Renpeng Xia, Xiang Zhang, Dan Su, Zhihua Li, Quanbo Zhou, Rufu Chen

**Affiliations:** 1 Department of Pancreas Center, Department of General Surgery, Guangdong Provincial People’s Hospital, Guangdong Academy of Medical Sciences, 510080 Guangzhou, Guangdong People’s Republic of China; 2 The Second School of Clinical Medicine, Southern Medical University, 510515 Guangzhou, Guangdong People’s Republic of China; 3 Guangdong cardiovascular Institute, 510080 Guangzhou, Guangdong People’s Republic of China; 4 Department of Pancreatobiliary Surgery, Sun Yat-sen Memorial Hospital, Sun Yat-sen University, 510120 Guangzhou, Guangdong People’s Republic of China; 5 Guangdong Provincial Key Laboratory of Malignant Tumor Epigenetics and Gene Regulation Medical Research Center, Sun Yat-sen Memorial Hospital, Sun Yat-sen University, 510120 Guangzhou, People’s Republic of China; 6 Department of Hepatobiliary, Pancreatic and Splenic surgery, The Sixth Affiliated Hospital of Sun Yat-sen University, 510655 Guangzhou, Guangdong People’s Republic of China; 7 Department of Neonatal/General Surgery, Hunan Children’s Hospital, 410007 Changsha, Hunan People’s Republic of China; 8 Department of Oncology, Sun Yat-sen Memorial Hospital, Sun Yat-sen University, Guangdong 510120 Guangzhou, People’s Republic of China; 9 School of medicine, South China University of Technology, 510006 Guangzhou, Guangdong province People’s Republic of China

**Keywords:** circCUL2, Inflammatory CAF, Pancreatic ductal adenocarcinoma, MyD88, NF-κB

## Abstract

**Background:**

Pancreatic ductal adenocarcinoma (PDAC) is characterized by clusters of cancer cells surrounded by a dense desmoplastic stroma. However, little is known about stromal cell heterogeneity in the pancreatic tumor microenvironment.

**Methods:**

We conducted circRNA profiling in primary fibroblasts by high-throughput sequencing and detected circCUL2 levels in PDAC tissues by qRT–PCR. We subsequently investigated the effect of circCUL2 on inflammatory cancer-associated fibroblast (iCAF) activation, heterogeneity and protumor activity by ELISA, flow cytometry, colony formation and transwell assays in vitro and by xenograft models in vivo. The regulatory effect of circCUL2 on miR-203a-3p/MyD88/IL6 was examined by RNA pulldown, FISH, and luciferase reporter assays.

**Results:**

We identified that circCUL2 was specifically expressed in cancer-associated fibroblasts (CAFs) but not in cancer cells. Moreover, the enrichment of circCUL2 in tumor tissues was significantly correlated with the poor prognosis of PDAC patients. Upregulation of circCUL2 expression in normal fibroblasts (NFs) induced the iCAF phenotype, and then iCAFs promoted PDAC progression through IL6 secretion in vitro. Furthermore, circCUL2-transduced NFs promoted tumorigenesis and metastasis of PDAC cells in vivo, which was blocked by an anti-IL6 antibody. Mechanistically, circCUL2 functioned as a ceRNA and modulated the miR-203a-3p/MyD88/NF-κB/IL6 axis, thereby further activating the STAT3 signaling pathway in pancreatic cancer cells to induce PDAC progression.

**Conclusions:**

We showed that the circCUL2/miR-203a-5p/MyD88/NF-κB/IL6 axis contributes to the induction of iCAFs and established a distinct fibroblast niche for PDAC progression, which could help the development of strategies that selectively target tumor-promoting CAFs in PDAC.

**Supplementary Information:**

The online version contains supplementary material available at 10.1186/s13046-021-02237-6.

## Background

Pancreatic ductal adenocarcinoma (PDAC), which has the worst prognosis of human malignancies [1], is poorly responsive to therapies and is histologically characterized by clusters of cancer cells surrounded by a dense desmoplastic stroma [2]. The tissue stroma undergoes transformation during cancer progression, and the interplay between cancer cells and the stroma creates a favorable tumor microenvironment (TME) for successful tumor development [3]. Previous efforts to deconstruct the desmoplastic stroma have been unsuccessful, which reflects the multifaceted nature of the TME, but a breakthrough could be achieved by exploring more biologically integrated targets to reshape the TME in PDAC.

Cancer-associated fibroblasts (CAFs) have long been considered to contribute most to malignant changes in the stroma by laying down extensive extracellular matrix around the tumor cells and secreting trophic factors that enable the formation of a protumorigenic niche [4]. CAFs are generally spindle shaped and positive for several activated fibroblast markers, such as fibroblast activation protein (FAP), α-smooth muscle actin and platelet-derived growth factor (PDGF) receptor-α (PDGFRα) [5, 6]. FAP is the first target used to assess the effect of cancer treatment on CAF inhibition. The unfavorable results of a phase II clinical trial using sibrotuzumab (a humanized monoclonal antibody) to target CAFs in patients with colorectal cancer prevented further investigation of this inhibitor [7]. Equally disappointing results were reported by another phase II trial showing limited efficacy against advanced PDAC of a combination of an oral small molecule inhibitor of FAP (talabostat) and gemcitabine [8]. Faced with unsuccessful attempts to inhibit CAFs, a more radical approach with genetic deletion of activated fibroblasts was proposed, which actually led to a more aggressive phenotype and worse overall survival (OS), indicative of the perplexity of the stroma-tumor interaction [9–11] and emphasizing the urgent need to exploit the composition and function of the stroma in PDAC.

CAFs are derived from pancreatic stellate cells (PSCs), quiescent resident fibroblasts and bone marrow-derived mesenchymal stem cells (MSCs) through activation of multiple signaling pathways. The activation of PSCs is considered a very early event during PDAC tumorigenesis [12, 13]. Recently, similar to the diversity in CAF origins, the heterogeneity in CAF fate and function has also attracted great attention and has indicated the possibility of targeting a subpopulation of CAFs to control malignancies. Researchers have discovered two CAF phenotypes, classified as myofibroblastic CAFs (myCAFs) and inflammatory CAFs (iCAFs), based on the expression levels of α-SMA [14]. myCAFs, characterized by strong α-SMA expression, are mainly located in the periglandular region of the TME and induce desmoplasia by direct juxtacrine interactions with cancer cells. In contrast, significantly lower αSMA levels were found in iCAFs, which play critical roles in promoting tumor progression through a group of cytokines and chemokines [14]. Accordingly, Giulia et al. discovered that iCAFs were stimulated by IL1 and provided a protumorigenic niche through JAK/STAT activation and IL6 secretion and shifts in the myCAF/iCAF ratio might have differential effects on PDAC progression [15]. Subsequently, a third subtype of CAFs, characterized by MHC class II molecule expression, was identified, with the ability to present antigens to CD4+ T cells [16], suggesting that the phenotypic and functional diversity of CAFs is still far from clarified.

Circular RNAs (circRNAs) are an interesting class of noncoding RNAs due to their unique generation by a noncanonical splicing event called backsplicing and their characteristics of covalently closed structures and exonuclease resistance [17]. circRNAs have diverse biological functions by acting as microRNA (miRNA) or protein sponges and regulating protein function or translation into peptides [18]. circRNAs have a potential role in tumorigenesis and act as oncogenes or tumor suppressors in different types of tumors [19], including PDAC [20, 21]. However, circRNAs have not been elucidated in the context of the TME in PDAC. Here, we demonstrated that the upregulation of circCUL2 expression in fibroblasts mediated the conversion of normal tissue-associated fibroblasts (NFs) into iCAFs and contributed to CAF heterogeneity in PDAC by inducing the MYD88-dependent NF-kb signaling pathway. These findings shed light on the development of strategies to selectively target CAFs that support tumor growth.

## Methods

### Patients and clinical samples

Tumor samples were collected from 161 patients with PDAC who underwent surgical resection at the Sun Yat-sen Memorial Hospital and Guangdong Provincial People’s Hospital between 2012 and 2020. None of the patients had received radiotherapy, chemotherapy, immunotherapy or targeted therapy before surgery. Tumor staging was determined using the 8th edition of tumor-node-metastasis (TNM) system of the American Joint Committee on Cancer. The clinical features of the enrolled patients are showed in Table 1. Overall survival (OS) was measured as the time interval from the date of randomization to the date of death or last follow-up evaluation (December 2020), and disease-free survival (DFS) was defined as the time interval between the date of randomization to the date of first disease-free failure event. All patients provided informed consent, and all related procedures were performed with the approval of the Ethical Committee of the indicated hospitals.

**Table 1:** Correlation between circCUL2 expression level and clinicopathologic characteristics of PDAC patients

Characteristics	N of cases	circCUL2 expression level		
		**Low**	**High**	**p value** ^a^
**Total cases**	161	81	80	
**Gender**				0.529
Male	87	46	41	
Female	74	35	39	
**Age, mean ± SD, year**^b^		60.10 ± 9.29	58.46 ± 7.96	0.232
**Differentiation**				0.061
Well	11	7	4	
Moderate	88	50	38	
Poor	62	24	38	
**TNM stage (AJCC)** ^c^				<0.001^***^
I	31	25	6	
II	103	50	53	
III	27	6	21	
**Lymph-node metastasis**				<0.001^***^
Negative	61	48	13	
Positive	100	33	67	
**Perineural invasion**				0.641
Negative	21	12	9	
Positive	140	69	71	
**Long-term smoking**^d^				0.093
No	141	67	74	
Yes	20	14	6	
**High-fat diet**				0.269
No	138	72	66	
Yes	23	9	14	
**Chronic pancreatitis**				0.831
No	135	67	68	
Yes	26	14	12	
Abbreviations: SD = standard deviation; TNM = tumor node metastasis; AJCC = American Joint Committee on Cancer. ^a^ Chi-square test (except for age), ^*^ p < 0.05, ^**^ p < 0.01, ^***^ p < 0.001. ^b^ Unpair t test, ^*^ p < 0.05, ^**^ p < 0.01, ^***^ p < 0.001. ^c^ Patients were staged in accordance with the 8th Edition of the AJCC Cancer’s’ TNM Classification. ^d^ Long-term smoking (duration ≥ 29 years).				

### Primary cell isolation and culture

Cancer-associated fibroblasts (CAFs) and primary normal fibroblasts (NFs) were isolated from pancreatic ductal carcinoma and corresponding noncancerous tissues. Freshly isolated surgical resections were collected with informed consent from Sun Yat-sen Memorial Hospital and Guangdong Provincial People’s Hospital. For CAF isolation, carcinoma samples were cut (0.5 cm^3^) from the core part of PDAC tissues using a sterile scalpel. Some of the isolated specimens were used for histological examination to confirm the diagnosis, and the remaining tissues were minced and dissociated by collagenase digestion medium (DMEM/F12, collagenase digestion 125 units/mg, insulin 10 mg/mL, hydrocortisone 0.5 mg/mL, penicillin 100 U/mL and streptomycin 100 mg/mL) [22]. The sample was digested for 30 min then quenched in 10% FBS/DMEM. Then the dissociated tissues were incubated without shaking plated on a 6-cm dish for 10 min at 37 °C. The stromal cell-enriched supernatant was collected in a new tube and centrifuged at 250 g for 5 min. For NF isolation, similar anatomopathological techniques were used, freshly isolated normal adjacent pancreatic tissue were taken from areas at least 3 cm distal to primary PDAC tumor masses [23]. All samples were taken from surgical resections, not from intrasurgical biopsies. Primary fibroblasts were cultured in Dulbecco’s modified Eagle’s medium (DMEM, GBICO) with 15% fetal bovine serum (FBS, GIBCO) and 1% penicillin/streptomycin at 37 °C in humidified air with 5% CO_2_.

For human PDAC cell lines culture, PANC-1 and MiaPaCa-2 were purchased from American Type Culture Collection (ATCC, Manassas, VA). The cells were then cultured in DMEM medium supplemented with 10% fetal bovine serum at 37 °C and 5% CO_2_.

### Flow cytometry assay

For identification of fibroblast population, CD31-FITC (4,220,516, BD Biosciences, USA), CD45-PE/Cy7 (561,868, BD Biosciences, USA), CD326 (EPCAM)-PE (2,088,498, Invitrogen, USA) were used to distinguish fibroblast from T cells, endothelial cells and epithelial cells. PDGFRα (3174, CST) and α-SMA (ab32575, Abcam) were used to distinguish iCAF from myCAF.

### RNA isolation and quantitative real-time PCR (qRT–PCR)

Total RNA was isolated with TRIzol (Life, USA) and reverse-transcribed using the PrimeScript RT Reagent Kit (DRR037A, Takara, Japan). Then, cDNA was amplified by qRT–PCR on a Light Cycler 480 Detection System (Roche, Switzerland) using the TB Green Premix Ex TaqTM kit (RR820A, Takara, Japan) with GAPDH or U6 as an internal control. The 2^−∆∆CT^ method was used to calculate relative gene expression levels in cells.

For expression in tissues, the levels were first normalized to GAPDH expression via the ∆CT method. To analyze the clinical significance of circCUL2, miR-203a-3p, IL6 and Myd88, tissues from 161 PDAC patients were divided into two groups—the low and high expression groups. Samples with normalized expression levels (∆CT) of these genes less than or equal to the median value were classified as the low expression group, and those with levels greater than the median value were classified as the high expression group.

For absolute quantification, 1 × 10^6^ NFs and CAFs cells were used for copy number analysis. Absolute quantification was performed via 10 times gradient dilution of the reference standard. Based on the measured cycle threshold (CT) values of the standards and the known concentrations of standards, we built a standard curve for the log (copy number) and CT values. The standard curve was used to extrapolate the number of molecules of circCUL2 and miR-203-3p in NFs and CAFs.

All primers are listed in Supplementary Table S1.

### RNase R digestion and actinomycin D assay

For RNase R digestion assay, total RNA of NFs and CAFs were treated with or without 5 U/µg RNase R (RNR07250, Epicenter Technologies) and incubated at 37℃ for 30 min. For actinomycin D assay, or total RNA was treated with 2 µg/mL actinomycin D (Sigma, USA) for 0 h, 4 h, 8 h, 12 and 24 h. And qRT–PCR was used to detected circCUL2 and CUL2 expression levels. The experiments were performed three times.

### Plasmid construction and transfection

circCUL2 was cloned into the pCD-ciR vector by IGE (Guangzhou, China). The luciferase reporter plasmids of circCUL2, the MyD88 3’ untranslated region (UTR) and mutant luciferase reporters were synthesized by IGE (Guangzhou, China). siRNA and miRNA mimic or inhibitor were purchased from IGE (Guangzhou, China). Plasmids and oligonucleotides were transfected using Lipofectamine 3000 (Invitrogen, USA) according to the manufacturer’s protocol. The targeted sequences of oligonucleotides are provided in Supplementary Table S2.

### Conditioned medium derived from human NFs and CAFs

After transfected, NFs and matched CAFs were cultured in 25 mL culture flasks at the same density, respectively. The supernatants were harvested after 48 h and centrifuged to removed cell pellets, then the conditioned medium (CM) was stored at -80 °C or incubation with PANC-1 or MiaPaCa-2 for 48 h.

### 5-Ethynyl-20-deoxyuridine (EdU) assay

Cell proliferation was measured by the EdU assay using BeyoClick EdU-555 detection kits (Beyotime, Shanghai, China), according to the manufacturer’s instructions. PDAC cells with indicated treatments were seeded into 24-well plates, followed by incubating with 50 µM EdU for 2 h. Cells were then fixed using 4% paraformaldehyde and sealed with Apollo reaction cocktail and Hoechst 33,342 in order. All images were photographed with a fluorescent microscope.

### Colony formation assay

500 PDAC cells with indicated treatments were seeded into 6-well plates and cultured for 2 weeks. Then the colonies were fixed in 4% paraformaldehyde for 20 min, followed by staining with 0.1% crystal violet. Colonies were then manually counted. Three different independent experiments were performed.

### Wound healing assay

PDAC cells with indicated treatments were seeded into 24-well plates. After 24 h, each well was wounded with a 20 µl pipette tip. The cell migration was photographed with an inverted microscope at the time points of 0 and 36 h. Three different independent experiments were performed.

### Transwell assays

The pretreated cells were cultured with 200 µl of serum-free medium in the top chamber that had been loaded with or without Matrigel (Matrigel BD biosciences, NY, USA). And 600 µl complete medium was added to the bottom compartment. After incubation for 18 h, cells on the upper surface of the top chamber were removed, and invaded cells were fixed and stained by crystal violet. The number of invaded cells was counted and captured with a light microscope. Three different independent experiments were performed.

### Cytokine array

The Proteome Profiler Human XL Cytokine Array Kit (R&D Systems, ARY022B, USA) was used to assess the cytokines secreted by fibroblast according to the manufacturer’s instructions. In brief, 400 µl of the indicated NF medium was incubated with an array membrane overnight. Then, detection antibody cocktails and streptavidin-HRP were added. The cytokine dots on X-ray films were scanned.

### Enzyme-linked immunosorbent assay (ELISA)

Concentration of cytokines were determined by using the human IL6 ELISA Kit (SEKH-0013, Solarbio, China) according to the manufacturer’s instructions. In brief, 100 µl of indicated NF medium was incubated with plates at 37℃ for 90 min. Then detection antibody, streptavidin-HRP and TMB were added in order. The absorbance of each well was measured at 450 nm with the SPARK 10 M spectrophotometer (Tecan, Austria).

### Western Blotting

Protein was extracted from the cells using RIPA lysis buffer (CWBIO, China), followed by subjected to SDS-polyacrylamide gels and transferred to polyvinylidene difluoride membranes. Corresponding primary antibodies including MyD88 (1:1000, 4283, CST), STAT3 (1:1,000, 9139, CST), p-STAS3(Tyr705) (1:1,000, 9145, CST), p65 (1:1,000, 8242, CST), pp65(Ser536) (1:1,000; 3033, CST), IkBα (1:1,000, 4812, CST), p-IkBα(Ser32) (1:1,000, 2859, CST), FAP (1:1,000, 28,244, Abcam), GAPDH (1:1,000; 132,004, Absin) were added to the membrane. HRP-conjugated secondary antibodies were used. The immunoreactive bands were detected by ECL detection system (Millipore, Germany) and photographed by Chemi XT4.

### Isolation of cytoplasmic and nuclear RNA

Cytoplasmic and nuclear RNA of CAFs were isolated using NE-PER Nuclear and Cytoplasmic Extraction Reagents (Thermo Scientific, USA) according to the manufacturer’s instructions. Then, the ratio of cytoplasmic and nuclear was measured by qRT–PCR. U6 served as the nuclear control, and GAPDH served as the cytoplasmic control.

### Fluorescence in situ hybridization (FISH)

FISH was performed using a In Situ Hybridization Kit (Gene Pharma, Guangzhou, China) according to the manufacturer’s instructions. FAM-labeled circCUL2 and Cy3-labeled hsa-miR-203a-3p probes (Gene Pharma, Guangzhou, China) were hybridized with cells overnight at 37℃. All images were captured by confocal microscopy. The targeted sequences of probes are provided in Supplementary Table S2.

### RNA pull-down assay

The biotinylated probes were synthesized by IGE (Guangzhou, China). Approximately 1 × 10^7^ CAFs and NFs were collected and lysed, followed by incubation with the biotinylated probe or oligo probe overnight at 4℃. Streptavidin magnetic beads (Invitrogen, USA) were added and then incubated for 3 h. The RNA-beads were isolated by TRIzol and analyzed by qRT–PCR.

### Luciferase reporter assay

The circCUL2/MyD88 wild-type or mutant plasmids and miR-203a-3p mimic were co-transfected into CAFs cells. Then the transfected Cells were seeded into 96-well plates and luciferase activities were determined by dual-luciferase reporter assay system (Promega, USA) according to the manufacturer’s instructions.

### Animal experiments

Animal experiments were conducted according to guidelines approved by the Animal Experimental Research Ethics Committee of South China University of Technology. BALB/c nude mice aged 4 to 5 weeks were purchased from South China University of Technology.

For the lung metastasis model, 100 µL suspension of 5 × 10^6^ PANC-1 cells or MiaPaCa-2 stably expressing luciferase (luc-PANC-1/luc-MiaPaCa-2) in PBS were injected into the tail vein of BALB/c nude mice. The mice were randomly divided into three groups, which were injected with luc-PANC-1 or luc-MiaPaCa-2 incubated with conditioned medium from (1) NFs transduced with empty vector for 48 h, (2) NFs transduced with circCUL2 vector for 48 h, (3) NFs transduced with circCUL2 vector in the presence of IL6 neutralizing antibodies (50 ng/mL, MAB206, R&D) for 48 h. The mice were imaged with an In Vivo Imaging System (IVIS Lumina XR Series III) 30 days later. Lung tissues were resected and were examined for metastatic foci via hematoxylin and eosin (HE) staining.

For the orthotopic model, the mice were randomly divided into three groups: (1) luc-tumor cells/empty vector-transduced NFs treated with PBS every three days, (2) luc-tumor cells/circCUL2-transduced NFs treated with PBS every three days, and (3) luc-tumor cells/circCUL2 transduced NFs treated with neutralizing antibodies against IL6 (2 mg/kg) every three days. BALB/c nude mice were anesthetized with ketamine (100 mg/kg) and xylazine (10 mg/kg). A 1-cm left subcostal incision was made to expose the pancreas, and a 50-µL suspension of luc-tumor cells/indicated NFs (1:1, 1 × 10^6^) in PBS was injected with a 30-G needle. After orthotopic implantation, the incision was closed with monofilament sutures. The mice were imaged with an In Vivo Imaging System (IVIS Lumina XR Series III) 30 days later to assess primary tumor size and metastasis.

For patient-derived xenograft (PDX) mouse models, fresh PDAC samples obtained from 3 patients were cut into small pieces and then implanted into subcutaneous in 4-week-old NSG mice (F1). Xenografts were removed from from F1 mice, cut into small pieces and replanted to other mice (F2). When tumors reached about 1500 mm^3^, they were excised and cut again into small pieces and replanted to other mice (F3). Seven days after xenografts reached about 100 mm^3^, F3 mice were divided into three groups randomly (n = 5 per group) and treated respectively with: (1) in vivo-optimized si-Control (50 mg/kg, RiboBio, Supplementary Table S2), i.v. injection every three days for 3 weeks; (2) in vivo-optimized si-circCUL2 (50 mg/kg, RiboBio, Supplementary Table S2), i.v. injection every 3 days for 3 weeks; (3) neutralizing antibodies against IL6. Tumor volume (length × width^2^/2) was monitored every 6 days.

### RNA-seq

Total RNA was isolated and purified using TRIzol (Life, USA) following the manufacturer’s procedure. After the quality inspection of Agilent 2100 Bioanalyzer (Agilent, USA) and NanoPhotometer (Implen, Germany), ribosomal RNA was removed from 1 µg total RNA. VAHTS Universal V6 RNA-seq Library Prep Kit for Illumina (Vazyme, China) was used for lncRNA library construction following the manufacturer’s protocol. Each library was sequenced on an Illumina Novaseq 6000 (Illumina Corporation, USA) in 150PE mode following the vendor’s recommended protocol by Guangzhou Huayin Health Medical Group CO.,Ltd. (Guangzhou, China).

### Statistical Analysis

All experimental data were expressed as mean ± standard deviation (SD) using GraphPad Prism 8.0. The differences between parametric variables were determined by Student’s t-test or one-way analysis of variance (ANOVA), and nonparametric variables were determined by Mann-Whitney U test. Statistical significance of survival was estimated by Kaplan-Meier analysis and the log-rank test, and correlation analysis was performed by two-sided Pearson’s correlation. Multivariate analysis of the relative risk was performed by cox regression. Correlation analysis was examined with two-sided Pearson’s correlation. p<0.05 was used as an indicator of statistical significance.

## Result

### Identification and clinical significance of circCUL2 in human CAFs and PDAC tissue

**Fig. 1:**
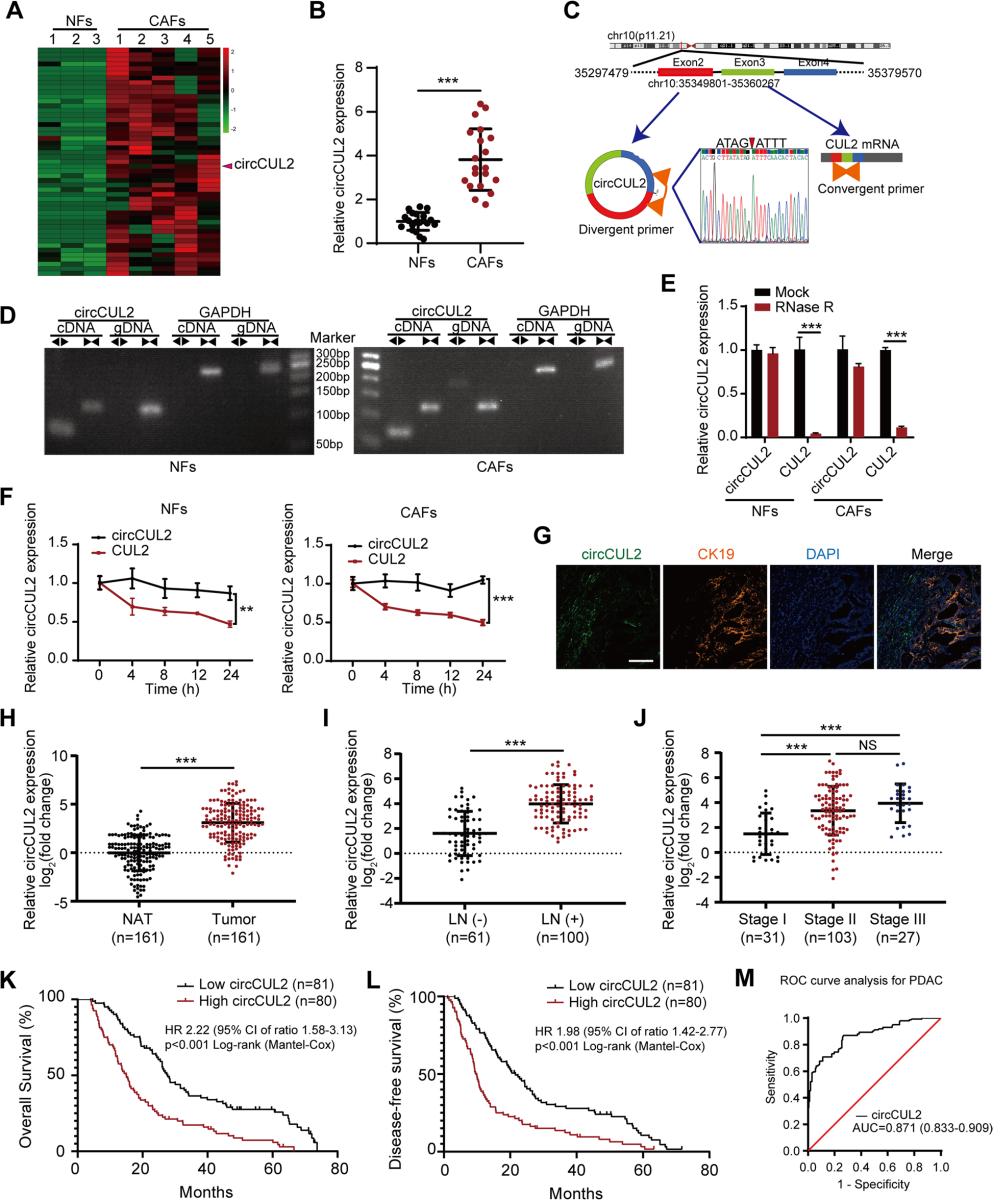
Identification of the circRNA circCUL2 in CAFs. **A** The cluster heat maps show the upregulated circRNAs in CAFs compared with NFs. circRNAs with fold change >2 and p < 0.05 were selected as significantly different. **B** qRT–PCR analysis of circCUL2 in 20 pairs of CAFs and matched NFs. **C** Schematic illustration showing the genomic location and splicing mode of circCUL2. The back-splice junction (red arrow) of circCUL2 was confirmed by sanger sequencing. **D** cDNA and gDNA of NFs and CAFs were amplified with convergent and divergent primers. GAPDH was as the negative control. **E** qRT–PCR analysis of circCUL2 and linear CUL2 in NFs and CAFs treated with RNase R. **F** qRT–PCR analysis of circCUL2 and linear CUL2 in NFs and CAFs treated with actinomycin D at the indicated time points. **G** localization of circCUL2 in pancreatic cancer tissue were detected by RNA FISH. CK19 indicated cancer cell. Scale bar, 50 μm. **H** The relative expression of circCUL2 in 161 PDAC tissue compared to paired NAT. **I**-**J **Association analysis between circCUL2 expression levels and LN status (**I**) and tumor stages (**J**). **K**-**L** Kaplan-Meier analysis of the correlation between circCUL2 expression levels and OS (**K**) and DFS (**L**) of PDAC patients. The median circCUL2 expression was used as the cutoff value. (M) ROC curve analysis of circCUL2 in PDAC tissue and normal tissues. Data are expressed as the mean ± SD of three independent experiments. ^**^p < 0.01 and ^***^p < 0.001 (two-tailed Student t-tests)

To identify critical circRNAs in fibroblast activation, we first isolated CAFs and NFs from human pancreatic cancer and paired normal pancreatic tissue. The characteristics of NFs and CAFs were confirmed by morphology, western blotting, immunofluorescence, and flow cytometry (Fig. S1A-D). Next, we conducted next-generation sequencing of five CAFs and three paired NFs (GSE172096) and identified 50 upregulated circRNAs in CAFs (Fig. 1 A). qRT–PCR analysis confirmed that circCUL2 (hsa_circ_0000234) was the most upregulated circRNA among the top 30 candidates (Supplementary Table S3) and was significantly upregulated by approximate 4-fold in CAFs compared with NFs (Fig. 1B). circCUL2 was generated from exons 2, 3 and 4 of the CUL2 gene with a length of 339 nt according to circBase (http://www.circbase.org/). Analysis of the circRNADb database predicted that the protein-coding potential of circCUL2 is relatively low. We verified the back-spliced junction of the reverse transcription product of circCUL2 by Sanger sequencing (Fig. 1 C). Furthermore, gel electrophoresis showed that circCUL2 could be amplified by divergent primers only from cDNA but not from genomic DNA (Fig. 1D). RNase R and actinomycin D treatment showed that circCUL2 has stronger resistance to RNase R and a lower degradation rate than CUL2 mRNA (Fig. 1E-F).

RNA FISH of circCUL2 in clinical PDAC tissue revealed that circCUL2 was located in the stroma and not in cancer cells (Fig. 1G). Accordingly, the expression of circCUL2 in the stroma was equal to that in the whole tumor tissue. Then, we analyzed the expression level of circCUL2 in a cohort of 161 PDAC patients. The expression of circCUL2 in PDAC tissues was significantly higher than that in paired normal adjacent tissues (NATs) (Fig. 1 H). Analysis of clinicopathological parameters based on circCUL2 levels revealed that high expression of circCUL2 was positively correlated with lymph node metastasis and late clinical stage (Fig. 1I-J; Table 1). In addition, Kaplan-Meier analysis indicated that PDAC patients with high circCUL2 levels had poorer OS and DFS (Fig. 1 K-L). Moreover, multivariate analysis revealed that the circCUL2 level and long-term smoking were independently correlated with OS and DFS (Tables 2 and 3). Receiver operating characteristic (ROC) curve analysis revealed that circCUL2 has clinical value for diagnosis (Fig. 1 M). Collectively, our findings suggest that circCUL2, as a stable circRNA, may be associated with PDAC progression.

**Table 2:** Univariate and multivariate analysis of Overall Survival (OS) in PDAC patients (n = 161)

Variables	Characteristics	Univariate analysis			Multivariate analysis		
		**HR**	**95% CI**	**p value**	**HR**	**95% CI**	**p value**
**Age**	< 60 (ref)						
	≥ 60	1.030	0.745-1.425	0.857			
**Gender**	Male (ref)						
	Female	0.885	0.640-1.225	0.459			
**Differentiation**	Well (ref)						
	Moderate	1.877	1.084-3.251	0.059	1.996	0.951-4.191	0.068
	Poor	2.446	1.434-4.174	0.007^**^	2.331	1.075-5.055	0.032^*^
**TNM stage**	I (ref)						
	II	1.178	0.786-1.765	0.439	0.826	0.524-1.304	0.413
	III	1.926	1.069-3.470	0.011^*^	1.116	0.607-2.052	0.723
**Lymph node metastasis** ^a^	Negative (ref)						
	Positive	1.731	1.251-2.395	<0.001^***^			
**Perineural invasion**	Negative (ref)						
	Positive	1.554	0.999-2.418	0.087			
**Long-term smoking** ^b^	No (ref)						
	Yes	1.744	0.970-3.136	0.018^*^	2.102	1.271-3.476	0.004^**^
**High-fat diet**	No (ref)						
	Yes	1.343	0.818-2.203	0.191			
**Chronic pancreatitis**	No (ref)						
	Yes	1.459	0.892-2.387	0.081			
**circCUL2 expression**	Low (ref)						
	High	2.222	1.575-3.133	<0.001^***^	2.374	1.645-3.426	<0.001^***^
Abbreviations: HR = hazard ratio; 95% CI = 95% confidence interval; TNM = tumor node metastasis; ref = reference. ^a^ As lymph node metastasis is included in the TNM stage, we didn’t include it in the multivariate analysis. ^b^ Long-term smoking (duration ≥ 29 years). Cox regression analysis, * p < 0.05, ** p < 0.01, *** p<0.001.							

**Table 3:** Univariate and multivariate analysis of Disease-free Survival (DFS) in PDAC patients (n = 161)

Variables	Characteristics	Univariate analysis			Multivariate analysis		
		**HR**	**95% CI**	**p value**	**HR**	**95% CI**	**p value**
**Age**	< 60 (ref)						
	≥ 60	1.125	0.820-1.545	0.461			
**Gender**	Male (ref)						
	Female	0.874	0.637-1.200	0.402			
**Differentiation**	Well (ref)						
	Moderate	1.878	1.112-3.171	0.047^*^	1.688	0.862-3.306	0.127
	Poor	2.131	1.251-3.630	0.020^*^	1.824	0.903-3.688	0.094
**TNM stage**	I (ref)						
	II	1.279	0.867-1.886	0.236	0.996	0.642-1.544	0.986
	III	1.744	0.980-3.104	0.032^*^	1.142	0.629-2.075	0.662
**Lymph node metastasis** ^a^	Negative (ref)						
	Positive	1.627	1.185-2.234	0.002^**^			
**Perineural invasion**	Negative (ref)						
	Positive	1.269	0.824-1.955	0.310			
**Long-term smoking** ^b^	No (ref)						
	Yes	1.788	0.989-3.232	0.013^*^	2.151	1.305-3.545	0.003^**^
**High-fat diet**	No (ref)						
	Yes	1.395	0.845-2.304	0.136			
**Chronic pancreatitis**	No (ref)						
	Yes	1.471	0.899-2.407	0.073			
**circCUL2 expression**	Low (ref)						
	High	1.982	1.418-2.771	<0.001^***^	2.073	1.460-2.942	<0.001^***^
Abbreviations: HR = hazard ratio; 95% CI = 95% confidence interval; TNM = tumor node metastasis; ref = reference. ^a^ As lymph node metastasis is included in the TNM stage, we didn’t include it in the multivariate analysis. ^b^ Long-term smoking (duration ≥ 29 years). Cox regression analysis, * p < 0.05, ** p < 0.01, *** p<0.001.							

### circCUL2 is critical to inducing and maintaining CAF protumor properties

**Fig. 2:**
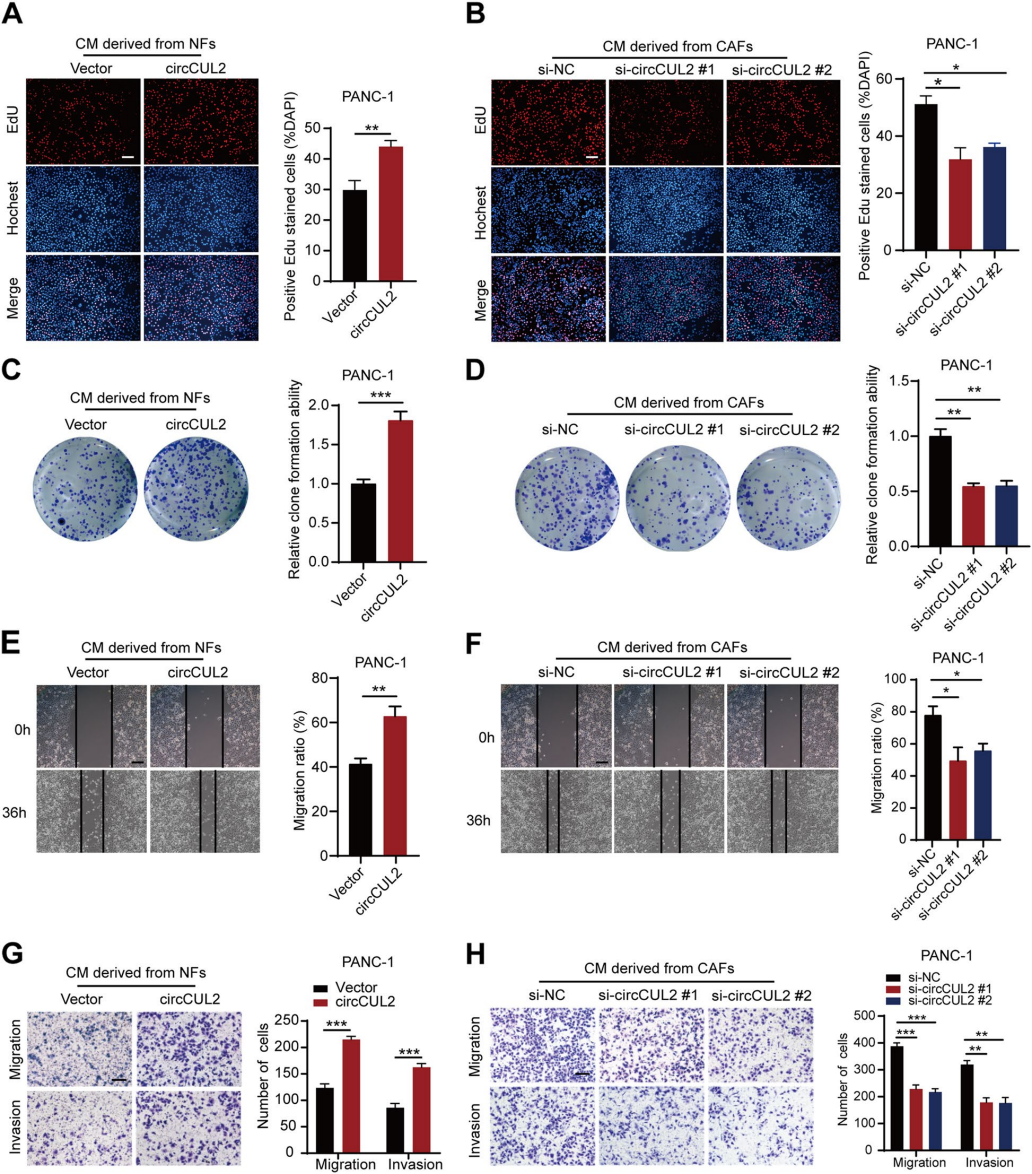
circCUL2 is critical to maintain CAFs pro-tumor activity in vitro. **A**-**B** EdU assay of the proliferation of PANC-1 cells treated with conditioned medium from circCUL2-overexpression NFs or circCUL2-silencing CAFs. Scale bar: 100 μm. **C**-**D** Colony formation assays in PANC-1 cells treated with conditioned medium circCUL2-overexpression NFs or circCUL2-silencing CAFs. **E**-**F** Scratch wound healing assays in PANC-1 cells treated with conditioned medium circCUL2-overexpression NFs or circCUL2-silencing CAFs. Scale bar: 100 μm. **G**-**H** Transwell assays of migration and invasion of PANC-1 cells treated with conditioned medium circCUL2-overexpression NFs or circCUL2-silencing CAFs. Scale bar: 100 μm. Data are expressed as the mean ± SD of three independent experiments. ^**^p < 0.01 and ^***^p < 0.001 (two-tailed Student t-tests)

To investigate whether circCUL2 affects the protumor activity of CAFs, we constructed the circCUL2 overexpression vector pcDNA3.1-circCUL2 and two siRNAs targeting the back spliced junction of circCUL2. The pcDNA3.1-circCUL2 vector was successfully transfected into NFs without increasing the mRNA and protein expression of the parental gene of circCUL2 (Fig. S2A-B). The two siRNAs were transfected into CAFs to knock down circCUL2 without altering CUL2 mRNA and protein expression (Fig. S2A-B). EdU assays and colony formation assays showed that conditioned medium from circCUL2-transduced NFs significantly enhanced the proliferation and cloning capabilities of PANC-1 cells and MiaPaCa-2 cells (Fig. 2 A, 2 C and Fig. S2C, 2E). Conditioned medium from circCUL2-silenced CAFs exerted the opposite effects (Fig. 2B and D and Fig. S2D, 2 F). Moreover, wound healing assays and transwell assays revealed that conditioned medium from circCUL2-transduced NFs significantly increased the migration and invasion ability of PANC-1 cells and MiaPaCa-2 cells (Fig. 2E and G and Fig. S2G, 2I), which considerably decreased upon silencing circCUL2 (Fig. 2 F, 2 H and Fig. S2H, 2 J). Besides, cells viability and apoptosis assays revealed that conditional medium from circCUL2-transduced NFs significantly enhanced gemcitabine resistance of PDAC cells, which reuduced upon silencing circCUL2 (Fig. S3A-D).These experiments indicated that circCUL2-transduced NFs displayed CAF protumor properties, while circCUL2-silenced CAFs lost their protumor properties.

### circCUL2-transduced NFs display an iCAF phenotype and promote PDAC progression by IL6

**Fig. 3:**
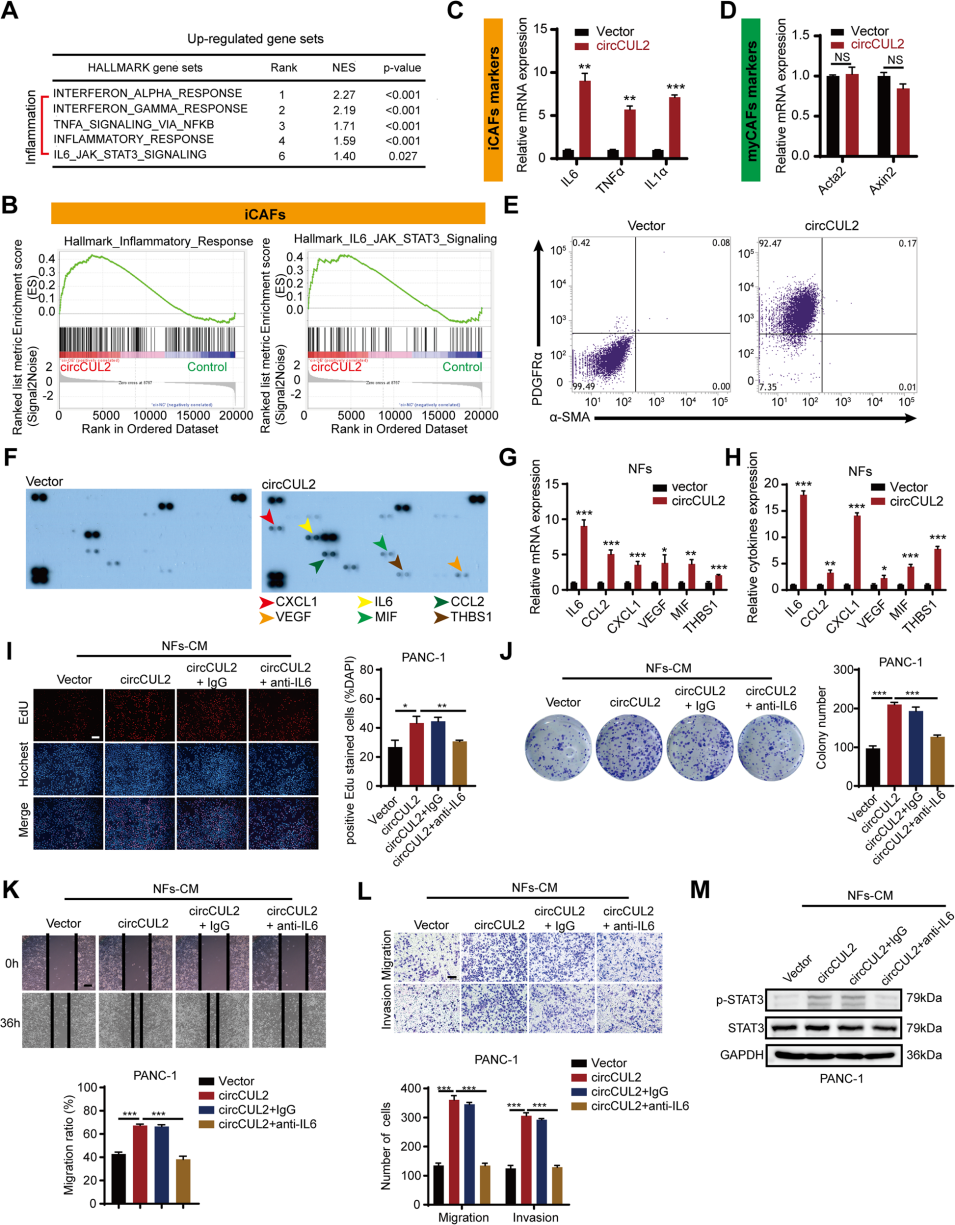
circCUL2 regulates the phenotypic plasticity of CAFs and promote PDAC progression by IL6. **A** Gene Set Enrichment Analysis (GSEA) of affected signatures in fibroblasts (circCUL2 vs. control). **B** GSEA plots for inflammatory CAF (iCAF) signatures in circCUL2 overexpression NFs from control. **C**-**D** qRT–PCR analysis of iCAF markers (IL6, TNF-α and IL1α) and myCAF marker (Acta2 and Axin2) expression in circCUL2 overexpression NFs. **E** Flow cytometric analysis of PDGFRα and α-SMA expression in circCUL2 overexpression NFs. **F**-**H**. Representative cytokine arrays for circCUL2-overexpression NFs and control (n = 3). arrows indicate the cytokines with significant changes, which were further confirmed by qRT–PCR and ELISA. **I**-**L** EdU assay (**I**), colony formation (**J**), Scratch wound healing assays (**K**) and transwell assays (**L**) of PANC-1 cells treated with conditioned medium circCUL2-overexpression NFs or anti-IL6. Scale bar, 100 μm. **M** western blot analysis of STAT3 and p-STAT3 in PANC-1 cells. Data are expressed as the mean ± SD of three independent experiments. ^**^p < 0.01 and ^***^p < 0.001 (two-tailed Student t-tests)

To clarify how circCUL2 confers NFs protumor properties, we performed mRNA sequencing of NFs and circCUL2-transduced NFs (GSE172272, Fig. S4A). KEGG and gene set enrichment analysis (GSEA) showed obvious enrichment signatures of inflammation in circCUL2-transduced NFs, which was consistent with the iCAF subtype identified by Öhlund et al. (Fig. 3 A-B, Fig. S4B-C) [14]. Furthermore, qRT–PCR showed that the expression of iCAF markers (IL6, TNFα and IL1α), but not myCAF markers (Acta2 and Axin2), was induced in circCUL2-transduced NFs (Fig. 3 C-D). Flow cytometry assays showed that the iCAF surface marker PDGFRα was overexpressed in circCUL2-transduced NFs, while the expression of the myCAF marker α-SMA did not change (Fig. 3E).

In addition, we performed a cytokine array to compare the cytokine profiles of NFs and circCUL2-transduced NFs. The results showed that a panel of cytokines were abundantly secreted by the circCUL2-transduced NFs, which was further confirmed by qRT–PCR and ELISA (Fig. 3 F-H). Among them, IL6 showed the strongest increase. EdU, colony formation, wound healing and transwell assays revealed that treating conditioned medium from circCUL2-transduced NFs with neutralizing antibodies against IL6 greatly abrogated the protumor activity of circCUL2-transduced NFs in vitro. Consistent with these findings, the antagonist IL6 in circCUL2-transduced NFs almost completely abrogated their effects on promoting the proliferation and invasion of tumor cells (Fig. 3I-L, Fig. S4D-G). As p-STAT3 is the most common target of IL6 [13, 24], we performed western blotting and found upregulation of p-STAT3 expression in PANC-1 cells and MiaPaCa-2 cells treated with conditioned medium from circCUL2-transduced NFs (Fig. 3 M, Fig. S4H). These results demonstrated that circCUL2 activated NFs into the iCAF subtype and promoted the progression of PDAC by greatly enhancing IL6 secretion.

### circCUL2-transduced NFs promote tumorigenesis and metastasis of PDAC cells in vivo

**Fig. 4:**
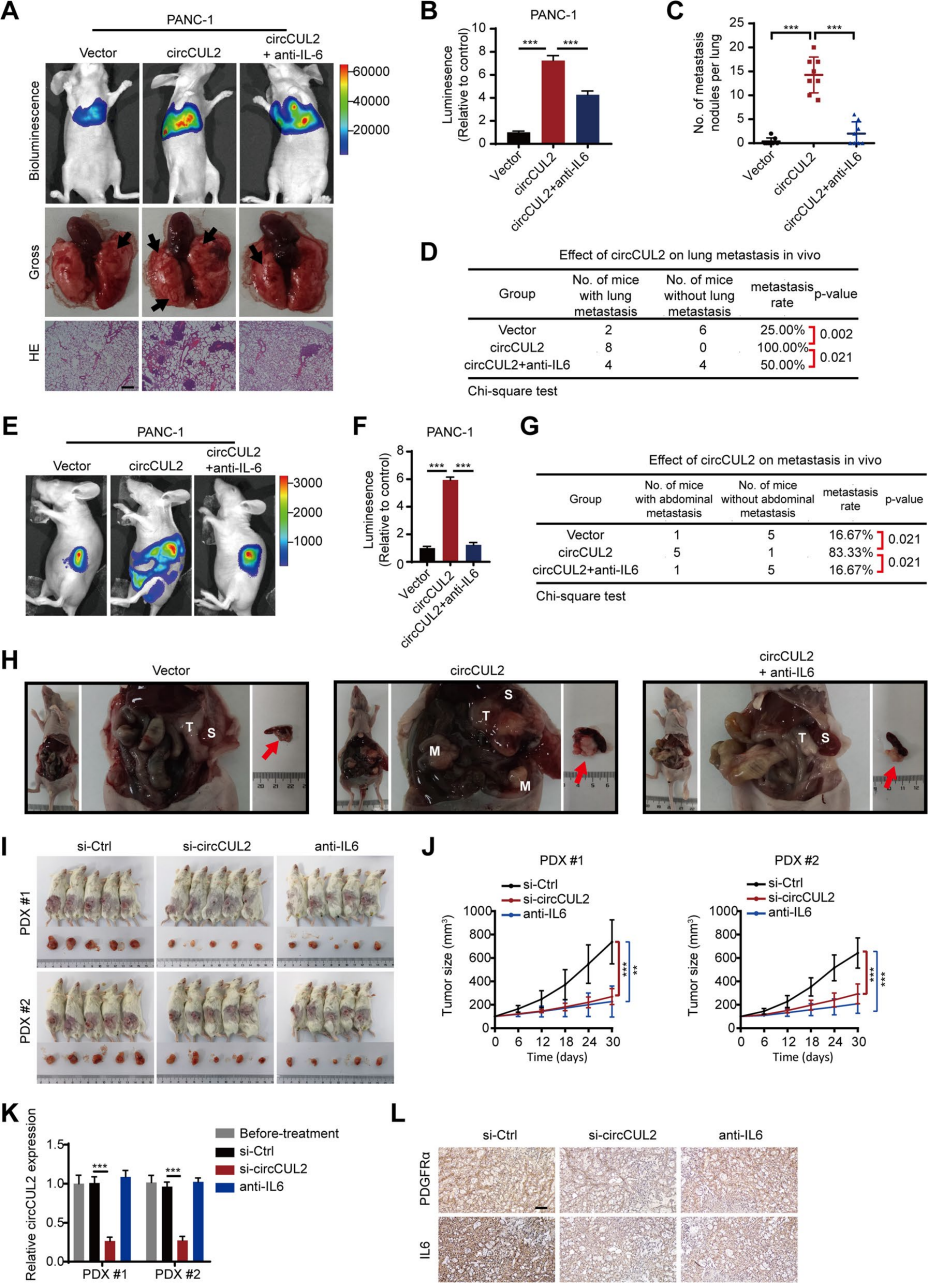
circCUL2-overexpression NFs promote PDAC progression in vivo. **A** Representative Bioluminescence images, lung and HE staining of lung tissue of mice 4 weeks after tail vein injection of luc-PANC-1 cell treated with conditioned medium as indicated (n = 8 per group). Scale bar, 100 μm. **B** Relative luminescence intensity in each group. **C** Histogram analysis of the metastatic nodules number in per lung. **D** lung metastasis rate of each group (Chi-square test). **E**-**F** Representative bioluminescence images and histogram analysis of luminescence intensity in each at day 30 are shown (n = 6). **G** Abdominal metastasis rate was calculated for indicated group (Chi-square test). **H** Representative images of orthotopic model in each group on which autopsy was performed. Red arrow indicated primary tumor; S, spleen; T, primary tumor; M, metastasis. **I** Images of PDX from 2 patients in 5 mice. (**J**) Tumor growth curves of indicated group (n = 5). **K** qRT–PCR analysis of circCUL2 levels in PDX of mice before and after treatment. **L** Representative images of IHC for PDGFRα and IL6. Scale bar, 100 μm. Data are expressed as the mean ± SD. ^**^p < 0.01 and ^***^p < 0.001 (two-tailed Student t-tests)

To determine the role of circCUL2-transduced NFs in the tumorigenesis and metastasis of PDAC cells in vivo, we constructed two different mouse models: a lung metastasis model and an orthotopic xenograft model. For lung metastasis model construction, luc-PANC-1 cells or luc-MiaPaCa-2 cells were collected after 48 h of in vitro coculture with empty vector- or circCUL2-transduced NFs. The intravital fluorescence imaging results revealed that the fluorescence intensity in the lung was markedly increased in the circCUL2 group compared with the control group (Fig. 4 A-B, Fig. S5A-B). Moreover, more lung metastatic foci and higher metastatic incidence were present in the circCUL2 group than in the control group (Fig. 4 C-D, Fig. S5C-D). Notably, adding a neutralizing antibody against IL6 to the coculture system of circCUL2-transduced NFs and tumor cells inhibited cancer cell metastasis (Fig. 4 A-D, Fig. S5A-D). For the orthotopic xenograft model, luc-PANC-1 cells or luc-MiaPaCa-2 cells were co-injected with empty vector- or circCUL2-transduced NFs into the pancreas of mice (Fig. S5E). The results revealed that circCUL2-transduced NFs significantly increased tumorigenesis compared with the control cells (Fig. 4E-F, Fig. S5F-G). Furthermore, a higher abdominal metastasis rate was presented in the circCUL2 group than in the control group (Fig. 4G-H, Fig. S5H-I), while treatment with the anti-IL6 antibody significantly inhibited tumor growth and abdominal metastasis (Fig. 4E-H, Fig. S5F-I).

To model the real tumor microenvironment of PDAC patients, we established a PDX model and performed treatment with a neutralizing antibody against IL6 or in vivo-optimized si-circCUL2 (Fig. S5J-L). We found that treatment with si-circCUL2 and anti-IL6 antibody significantly reduced PDX growth (Fig. 4I-J). qRT–PCR and IHC analysis showed that treatment with si-circCUL2 significantly repressed circCUL2 and IL6 levels. (Fig. 4 K-L). Collectively, these results indicate that circCUL2 plays an important role in PDAC tumorigenesis and metastasis by activating the iCAF phenotype and the production of IL6.

### circCUL2 functions as a miR-203a-3p sponge in fibroblast cells

**Fig. 5:**
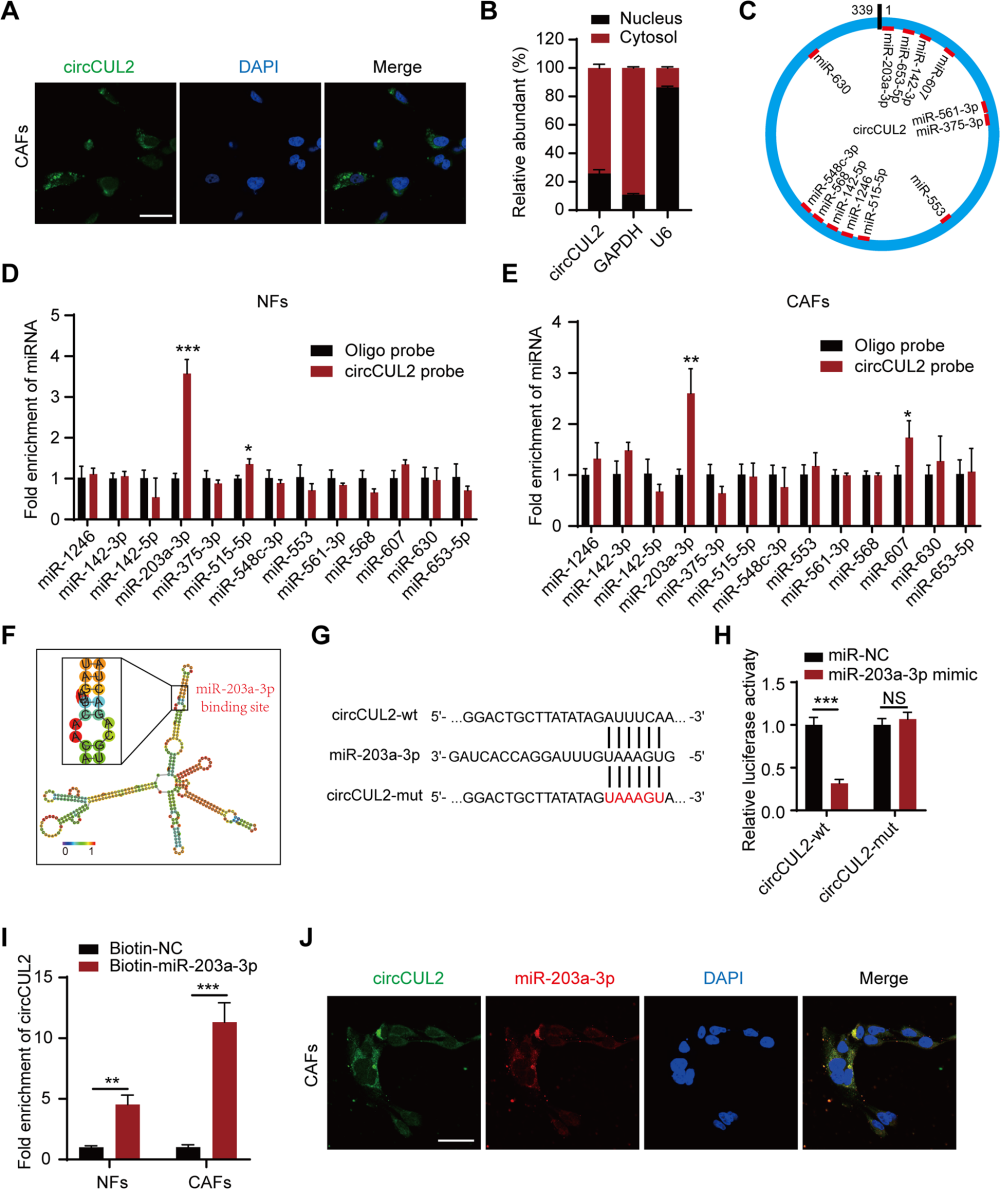
circCUL2 is a sponge of miR-203a-3p. **A** Subcellular localization of circCUL2 in CAFs detected by FISH. Scale bar, 50 μm. **B** circCUL2 expression in cytoplasm and nucleus of CAFs was detected by qRT–PCR. GAPDH and U6 RNA were used as cytoplasmic and nuclear RNA markers, respectively. **C** Schematic illustration of the potential target miRNAs of circCUL2 predicted by CircInteractome. **D**-**E** qRT–PCR analysis of the indicated miRNAs enriched with biotin-labeled circCUL2 probes in NFs and CAFs. **F** Diagram of the secondary structure of circCUL2 and the possible binding sites with miR-203a-3p predicted by RNAalifold. **G**-**H** Luciferase reporter assay was used to detect the luciferase activity of circCUL2-WT and circCUL2-mut (miR-203a-3p binding site mutated) luciferase reporter cotransfected with miR-203a-3p mimic. N.S., no significant. **I** qRT–PCR analysis of circCUL2 enriched with biotin-labeled miR-203a-3p probes in NFs and CAFs. **J** Subcellular localization of circCUL2 and miR-203a-3p in CAFs detected by FISH. Scale bar, 50 μm. Data are expressed as the mean ± SD. ^**^p < 0.01 and ^***^p < 0.001

Given that the functions of circRNAs are usually associated with their subcellular localization, we next performed FISH and subcellular fractionation assays to determine the localization of circCUL2 in CAFs. The results showed that circCUL2 was predominantly distributed in the cytoplasm, which indicated that circCUL2 may act as a miRNA sponge (Fig. 5 A-B). To explore the potential miRNAs bound to circCUL2, circInteractome was used, which predicted thirteen miRNAs (Fig. 5 C). Among these candidate miRNAs, only miR-203a-3p was enriched by circCUL2 in both NFs and CAFs (Fig. 5D-E). RNAalifold (http://rna.tbi.univie.ac.at/) was used to predict the binding site between circCUL2 and miR-203a-3p (Fig. 5 F), and a dual-luciferase reporter assay further confirmed that miR-203a-3p bound to circCUL2. The results of the assay revealed that miR-203a-3p mimics markedly decreased the luciferase activity of the circCUL2-wild-type (circCUL2-wt) group compared to the miR-NC group but had no effect on the luciferase activity of the circCUL2-mutant (circCUL2-mut) group (Fig. 5G-H). Accordingly, biotin-labeled miRNA pulldown assays validated the absorption of circCUL2 and miR-203a-3p (Fig. 5I). In addition, miRNA pulldown assays indicated that miR-203a-3p could not bind linear CUL2, and miR-203a-3p did not affect the mRNA and protein levels of linear CUL2 (Fig. S6A-C). Furthermore, FISH assays demonstrated the colocalization of circCUL2 and miR-203a-3p in the cytoplasm (Fig. 5 J). To support that circCUL2 could function as a ceRNA to sponge miR-203a-3p, the copy numbers of circCUL2 and miR-203a-3p were detected by absolute quantification in NFs and CAFs, which were at the same order of magnitude (Fig. S6D-E). These results suggest that circCUL2 functions as a miR-203a-3p sponge.

**Fig. 6:**
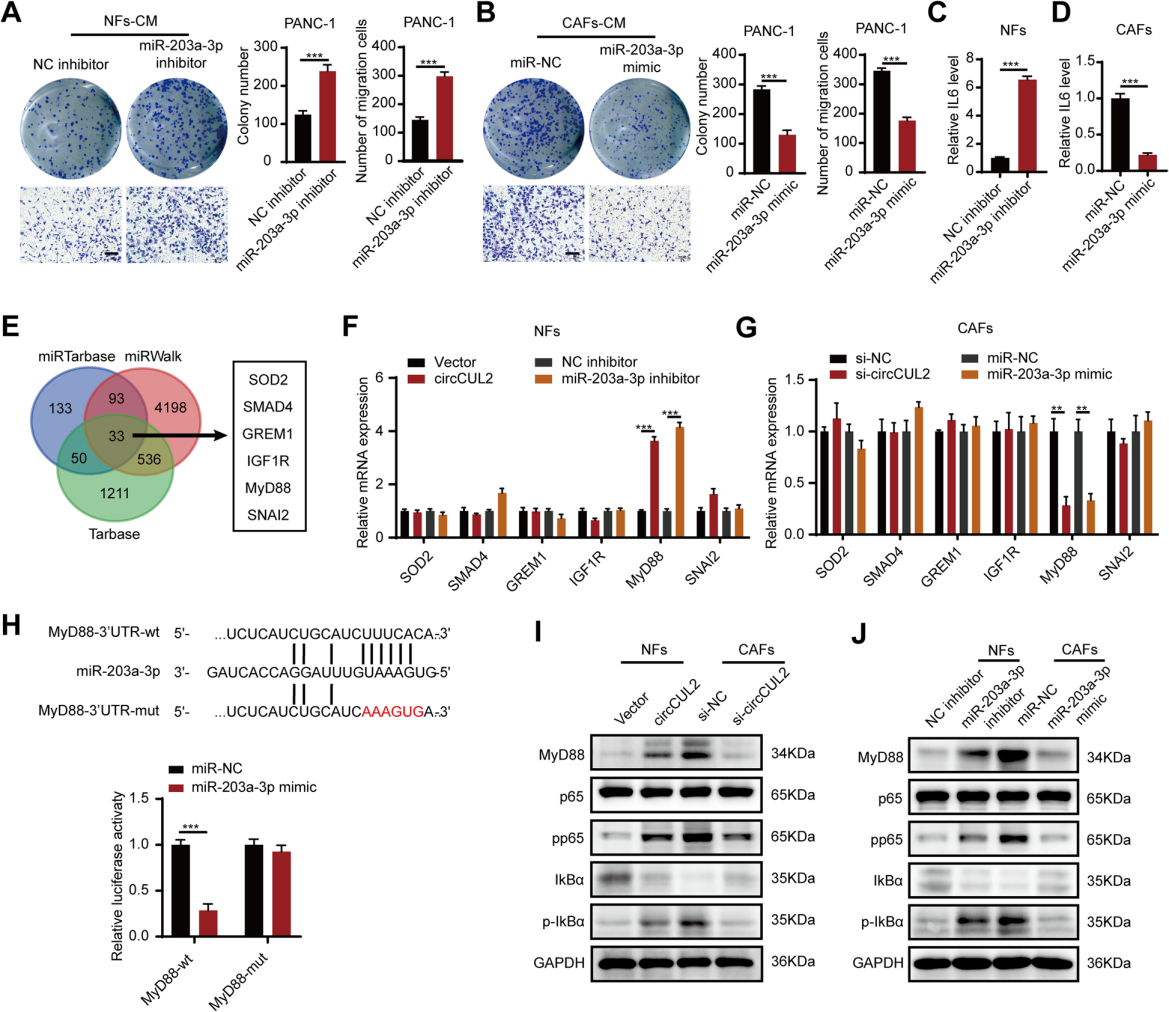
MyD88 is a direct downstream target gene of miR-203a-3p. **A**-**B** Colony formation and transwell assays of PANC-1 cells treated with conditioned medium from miR-203a-3p-silencing NFs or miR-203a-3p-overexpression CAFs. Scale bar: 100 μm. **C**-**D** ELISA assays detected IL6 level of conditioned medium from miR-203a-3p-silencing NFs or miR-203a-3p-overexpression CAFs. **E** Venn analysis of the potential downstream target genes of miR-203a-3p, predicted by miRTarbase, miRWalk and Tarbase. **F**-**G** qRT–PCR analysis of screened downstream target genes of miR-203a-3p in indicated NFs and CAFs. **H** Luciferase reporter assay was used to detect the luciferase activity of MyD88-Wild Type (MyD88-wt) and miR-203a-3p binding site mutated MyD88 (mYD88-mut) luciferase reporter cotransfected with miR-203a-3p mimic. N.S., no significant. **I**-**J** Western blotting analysis of MyD88, p65, pp65, IKBα and p-IKBα expression after overexpression of circCUL2 or silence of miR-203a-3p in NFs, or silence of circCUL2 or overexpression of miR-203a-3p in CAFs. GAPDH as a loading control. Data are expressed as the mean ± SD. ^**^p < 0.01 and ^***^p < 0.001

To verify the biological functions of miR-203a-3p, we transfected a miR-203a-3p inhibitor into NFs and a miR-203a-3p mimic into CAFs. Clone formation and transwell assays revealed that incubation with conditioned medium from miR-203a-3p-knockdown NFs promoted the proliferation and migration ability of PANC-1 cells and MiaPaCa-2 cells, whereas incubation with conditioned medium from miR-203a-3p-overexpressing CAFs suppressed proliferation and migration (Fig. 6 A-B, Fig. S7A-B). Additionally, the miR-203a-3p inhibitor enhanced, whereas its mimic reduced, the production of IL6 (Fig. 6 C-D).

### MyD88 is a downstream target of miR-203a-3p

miRNAs can regulate gene expression by interacting with the 3’UTR of mRNA [25]. The downstream target genes of miR-203a-3p were predicted by miRTarBase, miRWalk, and TarBase, and six candidate genes were screened by functional analysis based on the NCBI database for further validation (Fig. 6E). qRT–PCR revealed that only MyD88 was regulated by both circCUL2 and miR-203a-3p (Fig. 6 F-G). We found that the 3’UTR of MyD88 harbored specific sequences complementary to miR-203a-3p (Fig. 6 H). Luciferase assays showed that miR-203a-3p mimics markedly decreased the luciferase activity of the MyD88 3’UTR-wild-type (MyD88-wt) reporter but not the MyD88 3’UTR-mutant (MyD88-mut) reporter (Fig. 6 H). MyD88 plays a key role in NF-κB signal transduction [26]. Western blot analysis showed that overexpression of circCUL2 or silencing of miR-203a-3p significantly increased the levels of MyD88 and its downstream signal transducer NF-κB in NFs, while silencing circCUL2 or overexpression of miR-203a-3p had the opposite effect in CAFs (Fig. 6I-J). These results suggested that MyD88 is a downstream target of miR-203a-3p.

### circCUL2 promotes proliferation and migration via the MyD88/NF-κB/IL6 axis

**Fig. 7:**
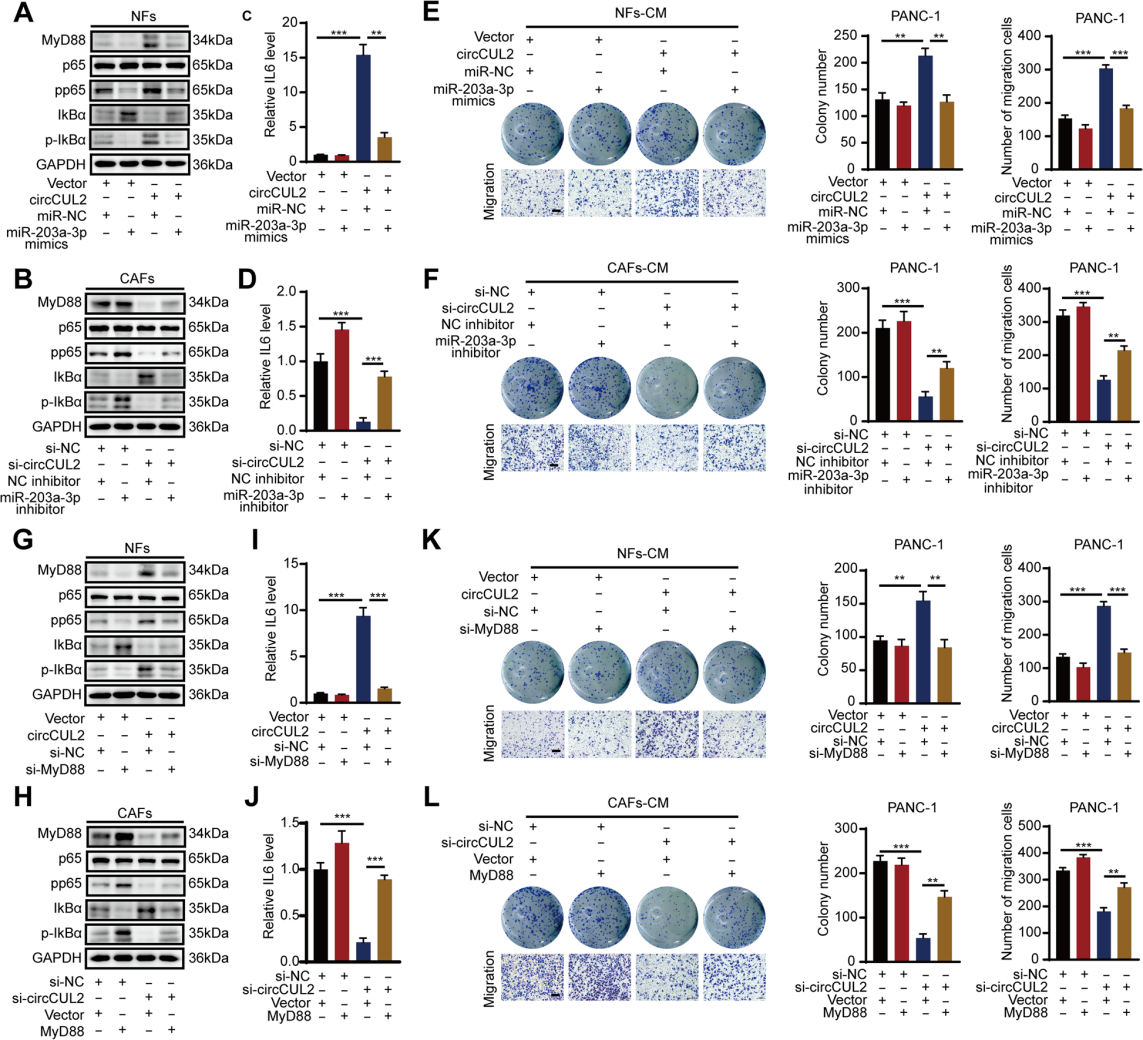
circCUL2 promotes proliferation and migration via MyD88/NF-κB/IL6 axis. **A**-**D** Cotransfection of circCUL2 overexpression plasmid and miR-203a-3p mimic in NFs or circCUL2 siRNA and miR-203-3p inhibitor in CAFs to detect the protein level of MyD88, p65, pp65, IKBα and p-IKBα, and secretion level of IL6. **E**-**F** PANC-1 cells were treated with conditioned medium from NFs cotransfected circCUL2 overexpression plasmid with miR-203a-3p mimic, or CAFs cotransfected circCUL2 siRNA and miR-203-3p inhibitor for 48 h. The proliferation and migration ability of PANC-1 were detected by colony formation and transwell assays. **G**-**J** Cotransfection of circCUL2 overexpression plasmid and MyD88 siRNA in NFs or circCUL2 siRNA and MyD88 overexpression plasmid in CAFs to detect the protein level of MyD88, p65, pp65, IKBα and p-IKBα and secretion level of IL6. **K**-**L** PANC-1 cells were treated with conditioned medium from NFs cotransfected circCUL2 overexpression plasmid with MyD88 siRNA, or CAFs cotransfected circCUL2 siRNA and MyD88 overexpression plasmid for 48 h. The proliferation and migration ability of PANC-1 were detected by colony formation and transwell assays. Data are expressed as the mean ± SD. ^**^p < 0.01 and ^***^p < 0.001

To clarify whether circCUL2 enhanced MyD88 expression by sponging miR-203a-3p, we performed rescue assays by cotransfection of a circCUL2 overexpression plasmid and miR-203a-3p mimic in NFs or silencing circCUL2 in the presence of the miR-203a-3p inhibitor. Western blot analysis showed that the promoting effect of circCUL2 overexpression on MyD88, pp65 and p-IkBα expression in NFs was abolished by the miR-203a-3p mimic, whereas the inhibitory effect of circCUL2 silencing in CAFs was reversed by the miR-203a-3p inhibitor (Fig. 7 A-B). Furthermore, ELISAs showed that the miR-203a-3p mimic markedly reversed the circCUL2 upregulation-induced increase in IL6 secretion in NFs, whereas the miR-203a-3p inhibitor significantly reversed the circCUL2 suppression-induced decrease in IL6 secretion in CAFs (Fig. 7 C-D). Similarly, colony formation and transwell assays revealed that the miR-203a-3p mimic significantly reversed the increase in cell proliferation and migration induced by circCUL2 overexpression in NFs, whereas the miR-203a-3p inhibitor reversed the suppression of cell proliferation and migration induced by circCUL2 depletion (Fig. 7E-F, Fig. S8A-B).

Next, we further tested whether the MyD88/NF-κB pathway was involved in the circCUL2-mediated progression of PDAC. Western blot analysis showed that forced expression of circCUL2 obviously increased the protein levels of p-65 and p-IkBα, whereas this effect was reversed by MyD88 suppression in NFs (Fig. 7G). Similarly, circCUL2 silencing significantly reduced the protein expression of p-65 and p-IkBα, and this effect was reversed by MyD88 overexpression in CAFs (Fig. 7 H). Moreover, ELISA revealed that MyD88 silencing strongly reversed the circCUL2 upregulation-induced increase in IL6 secretion in NFs, whereas MyD88 overexpression significantly reversed the circCUL2 suppression-induced decrease in IL6 secretion in CAFs (Fig. 7I-J). Accordingly, colony formation and t assays revealed that MyD88 silencing significantly reversed the increase in cell proliferation and migration induced by circCUL2 overexpression in NFs, whereas MyD88 overexpression reversed the suppression of cell proliferation and migration induced by circCUL2 depletion (Fig. 7 K-L, Fig. S8C-D). Collectively, our data suggested that circCUL2 promoted the proliferation and migration of PDAC via the miR-203a-3p/MyD88/NF-κB/IL6 axis.

### Clinical implications of the circCUL2/miR-203a-3p/MyD88/IL6 axis in PDAC patients

**Fig. 8:**
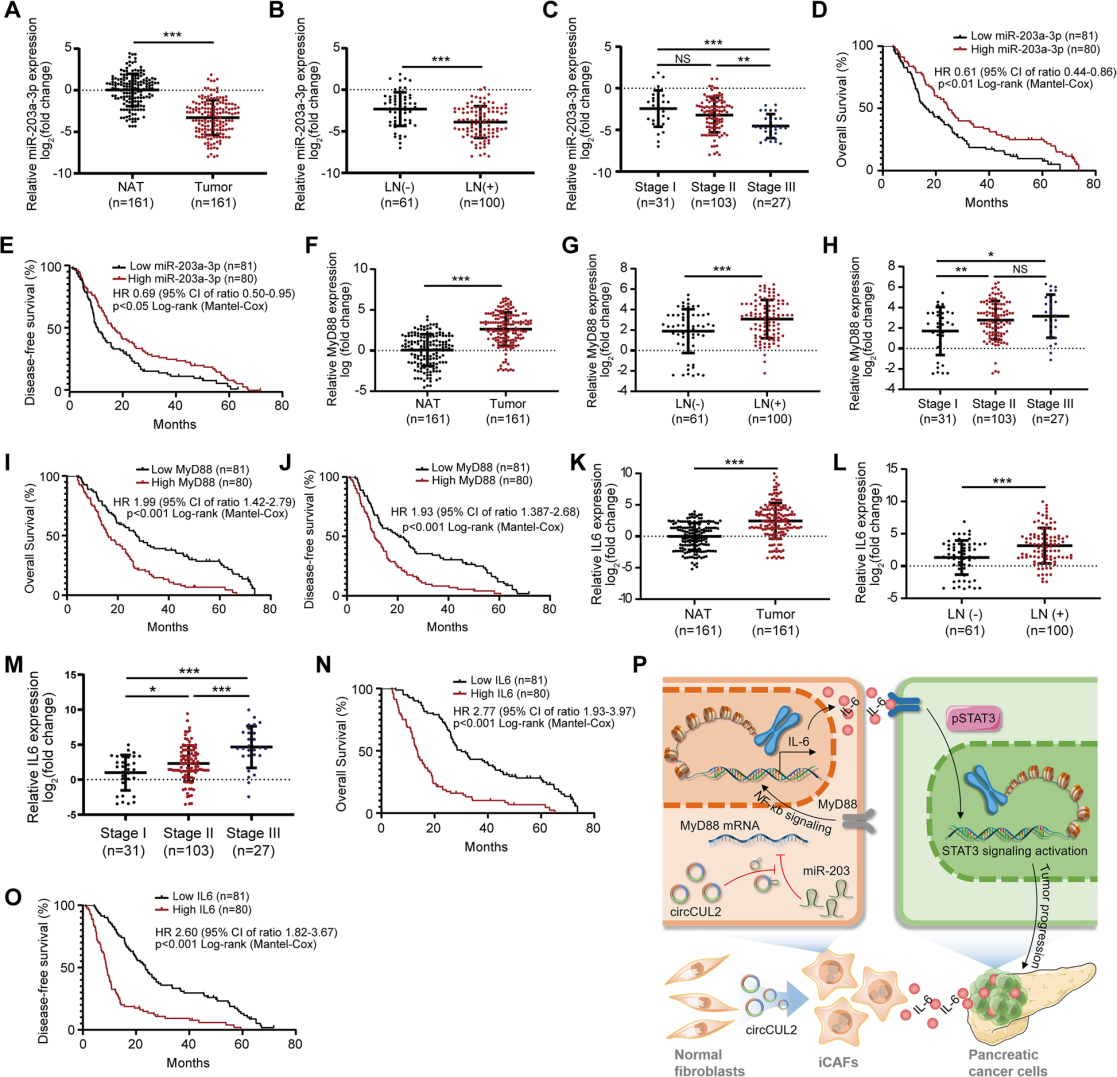
Clinical implication of circCUL2/miR-203a-3p/IL6 axis in PDAC. **A**, **F**, **K** The relative expression of miR-203a-3p (**A**), MyD88 (**F**) and IL6 (**G**) in 161 PDAC tissues compared to paired NAT. **B**-**C**, **G**-**H**, **L**-**M** Association analysis between miR-203a-3p (**B**-**C**), MyD88 (**G**-**H**) and IL6 (**L**-**M**) expression levels and LN status and tumor stages in 161 PDAC tissues. (**D**-**E**, **I**-**J**, **N**-**O**) Kaplan-Meier analysis of the correlation between miR-203a-3p (**D**-**E**), MyD88 (**I**-**J**) and IL6 (**N**-**O**) expression levels and OS or DFS of 161 PDAC patients. The median miR-203a-3p, MyD88 and IL6 expression was used as the cutoff value. **P** Proposed model indicates the mechanism by which circCUL2 activated iCAF phenotype and production of IL6 to promote malignant progression of PDAC via miR-203a-3p/MyD88/NF-κB pathway. Data are expressed as the mean ± SD. ^*^p < 0.05 and ^***^p < 0.001

Since circCUL2 regulates the miR-203a-3p/MyD88/IL6 axis in CAFs and promotes PDAC cell progression, we next analyzed the clinical relevance of this regulatory axis in a cohort of 161 PDAC patients and found that the expression of miR-203a-3p was lower in PDAC tissues than in NATs (Fig. 8 A). Low expression of miR-203a-3p was positively correlated with lymph node metastasis and late clinical stage (Fig. 8B-C). Kaplan-Meier analysis indicated that lower expression of miR-203a-3p resulted in poorer OS and DFS (Fig. 8D-E). Similarly, MyD88 expression was upregulated in PDAC tissues and high expression of MyD88 was positively correlated with lymph node metastasis and late clinical stage (Fig. 8 F-H). Kaplan-Meier analysis indicated that high expression of MyD88 resulted in poorer OS and DFS (Fig. 8I-J). Moreover, circCUL2 was negatively correlated with miR-203a-3p and positively correlated with MyD88 (Fig. S9A-B). Additionally, IL6 expression was upregulated in PDAC tissues and was associated with lymph node metastasis and late clinical stage (Fig. 8 K-M). Kaplan-Meier analysis revealed that higher expression of IL6 was accompanied by shorter OS and DFS (Fig. 8 N-O). Furthermore, IL6 was positively correlated with circCUL2 and negatively correlated with miR-203a-3p (Fig. S9C-D).

Collectively, our data support a model in which circCUL2 plays an important role in PDAC progression by activating iCAF production of IL6 via miR-203a-3p/MYD88/NF-kB signaling (Fig. 8P).

## Discussion

Due to the development of high-throughput sequencing technology and bioinformatics, circRNAs have attracted increasing attention in the noncoding RNA field and have been discovered and reported as critical regulators of various biological processes, including multiple cancers [27, 28]. Currently, the roles of circRNAs in the progression of pancreatic cancer have been revealed in many studies [29]. Recently, our team reported that circBFAR promotes the progression of PDAC via the miR-34b-5p/MET/Akt axis and that circNFIB1 inhibits lymphangiogenesis and lymphatic metastasis via the miR-486-5p/PIK3R1/VEGF-C axis in pancreatic cancer [20, 21]. However, little is known about the role and underlying mechanisms of circRNAs in the context of the TME in PDAC. Herein, we identified differentially expressed circRNAs in primary isolated fibroblasts and demonstrated that circCUL2 was highly expressed in iCAFs and that its enrichment in tumor tissues was correlated with the prognosis of patients with PDAC. Overexpression of circCUL2 in NFs drove the cells toward the iCAF phenotype, and then iCAFs promoted the progression of PDAC by secreting IL6. Mechanistically, circCUL2 functioned as a ceRNA and modulated the miR-203a-3p/MyD88/NF-κB/IL6 axis, thereby further activating the STAT3 signaling pathway in pancreatic cancer cells to induce the progression of PDAC. Regarding the function of circCUL2 in gastrointestinal tumors, circCUL2 was found to promote tumor malignancy and metastasis in hepatocellular carcinoma [30], while recently, it was revealed that it may function as a tumor suppressor and mediator of cisplatin sensitivity in gastric cancer [31]. Our study found that circCUL2 can promote the platinum resistance of pancreatic cancer through the activation of iCAFs, which is inconsistent with the conclusions of the recently finding in gastric cancer [31]. These findings indicate the heterogeneity of the expression and biological function of circCUL2 in different tissues in the context of the complexity of the TME. To our knowledge, this is the first report to provide insight into the biological significance of the circRNA-mediated phenotypic transition of iCAFs in PDAC and highlight that circCUL2 may serve as a novel therapeutic target for iCAFs that support tumor growth.

For years, the mechanisms of genetic and epigenetic modifications in pancreatic cancer cells have been deeply explored, while the complex TME is far from clear. circRNAs have recently attracted enormous attention, and their underlying cancer-associated mechanism in altering the TME is gradually being revealed. By performing a comprehensive bioinformatics analysis of one of the most studied circRNAs, CDR1as, Zou et al. showed that CDR1as may play a specific role in stromal and immune cell infiltration in tumor tissue and could promote cancer progression by altering the TME [32]. Recently, Kristensen et al. reported that CDR1as was undetectable in colon cancer cells in vivo but was abundantly expressed within tumor stromal cells, which highlights the importance of spatially resolving the expression patterns of circRNAs in the TME [33]. Determining the specific mechanisms that control the fate and function of CAFs in PDAC is needed for the development of therapeutic regimens that selectively target tumor-promoting CAFs. For the first time, we explored the potential involvement of circRNAs in primary fibroblasts from human PDAC tissues. Based on circRNA profiling in primary CAFs and NFs from human PDAC adjacent normal tissue by next-generation sequencing, we focused on circCUL2, and the high circCUL2 level in stoma was positively correlated with lymph node metastasis and advanced clinical stage and independently correlated with OS and DFS. At present, many studies have shown that the correlation between tumor gene expression and prognosis rarely provides in situ data to assess the potential intratumor heterogeneity of target gene expression. Especially in PDAC, which has a fibroblastic population comprising 90% of the whole tumor mass [34], the expression patterns of circRNAs in stromal cells are far from elucidated. Our study is the first to link circRNA expression in CAFs with the prognosis of PDAC patients, and the results indicated that the heterogeneity of gene expression in stromal cells also plays a key role in tumor progression, suggesting the potential of targeting circRNAs in the TME to improve therapeutic outcomes.

For the key role of circCUL2 in fibroblasts, our gain- and loss-of-function experiments demonstrated that circCUL2 was critical to maintaining CAF protumor properties and that the transduction of circCUL2 into NFs was sufficient to induce a distinct CAF subtype characterized by an inflammatory phenotype. Our GSEA of mRNA-seq in NFs and circCUL2-transduced NFs showed that the hallmarks of the inflammatory response and IL6/JAK/STAT3 signaling were both significantly enriched. Furthermore, both qRT–PCR and flow cytometry assays confirmed the activation of iCAF markers (IL6, TNFα, IL1α and PDGFRα) but not myCAF markers (Acta2, Axin2 and α-SMA). In addition, a panel of inflammatory cytokines abundantly secreted by circCUL2-transduced NFs was also confirmed by cytokine array and ELISA. Intriguingly, our data revealed a significantly negative enrichment of circCUL2 overexpression and hallmarks of myogenesis. Consistent with recent work from Biffi et al., IL1R1 expression leads to NF-κB/LIF/JAK/STAT activation and iCAF formation [15, 35]. Moreover, JAK inhibition or tumor-secreted TGF-β significantly increased the myCAF/iCAF ratio in treated tumors, further confirming that iCAFs and myCAFs are interconvertible cell states rather than endpoints in differentiation [15]. Considering the key role of the circCUL2-induced cascade in the activation of the NF-κB/IL6 signaling pathway, targeting circCUL2 could potentially convert iCAFs into a more myofibroblastic state that has been previously suggested to inhibit tumor progression.

Accumulating reports have revealed that circRNAs always function as efficient miRNA sponges to then modulate miRNA target gene expression. In our study, miR-203a-5p was selected as the candidate target miRNA of circCUL2, and the circCUL2/miR-203a-5p interaction was confirmed by FISH, RNA pulldown and luciferase reporter assays. Interestingly, several studies identified miR-203 as a matrix stiffness-repressed transcript [36–38], and the highly stiff ECM leads to a low miR-203 level in the TME, increasing breast cancer risk [38], which indicates the key role of miR-203 in mediating the crosstalk between cancer cells and stroma. Our study provided the first evidence that the downregulation of miR-203 expression in NFs leads to the activation and transition of the iCAF state and revealed specific signal transduction mediated by miR-203 in stromal cells.

Intersection of the prediction results of three databases identified MyD88 as the downstream target of miR-203a-3p, which was further verified by qRT–PCR, luciferase assays and 3’UTR mutation experiments. MyD88 plays a key role in NF-κB signal transduction and is involved in both oncogene-induced intrinsic and extrinsic inflammation in cells [39]. A large number of studies have shown that the adaptor protein MyD88 contributes to carcinogenesis, including cancer of the skin, liver, pancreas, and colon, by acting downstream of Toll-like receptors (TLRs) or the IL1 family [39]. Recently, Biffi et al. demonstrated a signaling cascade induced by the IL1 family that leads to NF-κB/JAK/STAT activation to generate iCAFs, and an IL1 receptor antagonist might be an efficient strategy to target iCAFs in vivo [15]. Most often associated with the IL1 family, MyD88 signaling mediates a proinflammatory feedback mechanism that is involved in the intrinsic inflammation associated with oncogene activation, cell transformation, and senescence [40]. Consistent with these findings, our study underlined the key role of MyD88 in activating the NF-κB/IL6 signaling cascade to mediate the transition of iCAFs. Although the abundance of cytokines, such as CCL18 and IL1b, in cancer cells may be responsible for initiating NF-kB signaling through activation of IKK in the CAF subset [41, 42], numerous feedback loops in the TME lead to the failure of therapeutic strategies to target inflammatory factors. Our study indicates a more meaningful strategy of antagonizing MYD88 endogenously by circCUL2.

As a classic proinflammatory pathway, NF-κB signaling has been implicated as a hallmark of cancer progression and a potential therapeutic target. In recent years, the role of NF-κB signaling in mediating the reciprocal interplay between cancer cells and stroma has gradually been revealed [41]. During the early preneoplastic stages of tumorigenesis, resting fibroblasts are activated to express a proinflammatory gene signature, thereby promoting cancer progression in an NF-κB-dependent manner [41]. Nearly 70% of PDAC cases show NF-κB activation [43], and several studies have revealed that NF-κB activation is required for the formation of iCAFs [14–16]. Our study provides novel results that the upregulation of circCUL2 expression in fibroblasts increases the expression of MyD88, the key receptor upstream of the NF-kb pathway, thereby contributing to the function of various proinflammatory factors from the TME in generating iCAFs.

In our study, we revealed that IL6 is the key downstream mediator of circCUL2. circCUL2- induced iCAFs contributed to the tumorigenesis and metastasis of PDAC through increased secretion of IL6 and further activation of the STAT3 signaling pathway in pancreatic cancer cells. Previous studies have revealed that IL6 secreted by iCAFs is involved in increasing proliferation and invasion of PDAC mouse cells [15, 44]. We reported for the first time that circCUL2 plays a key role in promoting the development of IL6-related iCAF phenotypes. Impressively, it has been gradually elucidated that IL6 secreted by CAFs mediates immunosuppression in pancreatic cancer. Recently, Thomas et al. demonstrated that IL6 blockade enhanced the efficacy of anti-PD-L1 therapy by promoting the T helper cell differentiation and increasing CD8+ T cell infiltration into PDAC tumors mice [45]. IL6 can also contribute to immunosuppression by promoting the differentiation of macrophages, and myeloid-derived suppressor cells and driving the apoptosis of type 1 conventional dendritic cells [46–48]. As circCUL2 contributes to iCAF phenotype development and the enrichment of the cytokine IL6 in pancreatic cancer, further exploration is needed to reveal its role in the immunosuppression of pancreatic cancer. Signal transduction downstream of IL6 includes STAT3, MAPK and PI3K carcinogenic pathways [13, 49, 50]. Marina et al. revealed that the IL6/Stat3 pathway is strongly activated and contributes to the progression of pancreatic intraepithelial neoplasia progression and the development of PDAC [13]. Zhang et al. reported that IL6 was able to synergize with Kras to facilitate the progression of pancreatic cancer precursor lesions by activating the MAPK signaling cascade [51]. Our data highlighted that the IL6/STAT3 pathway is the key mediator of the protumorigenic properties of iCAFs in PDAC, but we do not exclude the involvement of other pathways, because these downstream signaling pathways form a complex regulatory network to induce synergistic effects [52]. Although most recent studies emphasized the effect of IL6/STAT3 pathway [53], other signaling pathways in this network, including MAPK and PI3K, still need to be further explored.

For the clinical implications of the circCUL2/miR-203a-3p/MyD88/IL6 axis in PDAC patients, we revealed that high levels of circCUL2, MyD88 and IL6 expression in tumor tissues are associated with poor prognosis in patients with pancreatic cancer, and high miR-203a-3p expression indicates better OS and DFS in PDAC patients. By conducting correlation analyses on the circCUL2/miR-203a-3p/IL6 axis, a positive correlation was found between circCUL2 and IL6 (R = 0.42, P < 0.001) or MyD88 (R = 0.44, P < 0.001), and negative correlations were found between miR-203a-3p and circCUL2 (R = -0.43, P < 0.001) or IL6 (R = -0.34, P < 0.001). The R values indicate that these correlations are not strong (|R| < 0.60) [54], which implies that the clear significance based on the p value may be supported by a high number of samples. One limitation of this study is that the RNA levels of the genes were detected in tissue samples, not the corresponding primary fibroblasts, which may affect the accuracy of the correlation analysis results. Gene expression correlation analysis in primary isolated cells may provide stronger evidence for the prognostic value of the circCUL2/miR-203a-3p/MyD88/IL6 axis. The RNA expression level of circCUL2 in tissues can be used to differentiate the pancreatic cancer tissues and normal tissues to a certain extent. The clinical translational value of circCUL2 can be further explored by detecting the expression levels of circCUL2 in the serum of pancreatic cancer patients and healthy people.

## Conclusions

Altogether, our observations demonstrated that circCUL2 was highly expressed in iCAFs and that its enrichment in tumor tissues was correlated with the prognosis of patients with PDAC. circCUL2 functioned as a ceRNA and modulated the miR-203a-3p/MyD88/NF-κb/IL6 axis, inducing the conversion of NFs into iCAFs, thereby further activating the STAT3 signaling pathway in pancreatic cancer cells to induce the progression of PDAC. This is the first report to reveal the biological processes of circRNA-mediated iCAF phenotypic transition and establish a distinct fibroblast niche for the progression of PDAC. The discovery of the role of the circCUL2/MyD88/NF-κb signaling pathway in activating and maintaining iCAFs in the TME may be needed for the development of rational strategies that selectively target tumor-promoting CAFs in PDAC.

## Supplementary Information

**Additional file 1.**

**Additional file 2.**

**Additional file 3:** **Figure S1.** Isolation and identification of NFs and CAFs. (A) Morphology of NFs and CAFs isolated from clinical samples under light microscope. (B-C) Immunofluorescence and western blot analysis of FAP in NFs and CAFs isolated from clinical samples. Scale bar, 50 μm. (D) CAFs isolated from clinical samples were negative for EpCAM (epithelial marker), CD31 (endothelial marker) and CD45 (leukocyte marker), determined by flow cytometry. PANC-1 cancer cells, human umbilical vein endothelial cells (HUVEC) and human T lymphocytes were used as positive controls. Images for a representative sample were shown.

**Additional file 4.** 

**Additional file 5:** **Figure S2. **Specificity of circCU2L siRNA and overexpression vector, related to Fig. 2. (A) qRT–PCR analysis of circCUL2 and CUL2 expression following transfecting circCUL2 siRNA and overexpression vector inNFs and CAFs. (B) Western bolt analysis of CUL2 in NFs transfected with circCUL2 vector and in CAFs transfected with circCUL2 siRNA. (C-D) EdU assay of the proliferation of MiaPaCa-2 cells treated with conditioned medium from circCUL2-overexpression NFs or circCUL2-silencing CAFs. Scale bar: 100 μm. (E-F) Colony formation assays in MiaPaCa-2 cells treated with conditioned medium from circCUL2-overexpression NFs or circCUL2-silencing CAFs. (G-H) Scratch wound healing assays in MiaPaCa-2 cells treated with conditioned medium from circCUL2-overexpression NFs or circCUL2-silencing CAFs. Scale bar: 100 μm. (I-J) Transwell assays of migration and invasion of MiaPaCa-2 cells treated with conditioned medium from circCUL2-overexpression NFs or circCUL2-silencing CAFs. Scale bar: 100 μm. Data are expressed as the mean ± SD. ^**^p <0.01 and ^***^p < 0.001.

**Additional file 6:** **Figure S3. **circCUL2 confers oxiaplatin resistance to PDAC cells. (A-B) Cell viability assay in PANC-1 and MiaPaCa-2 cells treated with conditioned medium from circCUL2-overexpression NFs or circCUL2-silencing CAFs. (C-D) Apoptosis assay in PANC-1 cells treated with conditioned medium from circCUL2-overexpression NFs or circCUL2-silencing CAFs.

**Additional file 7:** **Figure S4. **circCUL2 activates iCAF phenotype, related to Fig. 3. (A)Volcano plots of different expression genes in circCUL2-transducted NFs and empty vector-transduced NFs. different expression genes were selected by p <0.05 and fold-change >2. Gray dots indicated genes without significantly different expression, red dot indicated genes significantly up-regulated, and green indicated genes significantly down-regulated. (B) Enrichment of KEGG Pathway of different expression genes associated with cancer in circCUL2-transducted NFs. (C) GSEA plots for inflammatory CAF (iCAF) and myofibroblast-like CAF (myCAF) signatures in circCUL2 overexpression NFs, compared with control. (D-G) EdU assay (D), colony formation (E), Scratch wound healing assays (F) and transwell assays (G) of MiaPaCa-2 cells treated with conditioned medium from circCUL2-overexpression NFs or anti-IL6. Scale bar, 100μm. (H) western blot analysis of STAT3 and p-STAT3 in MiaPaCa-2 cells. Data are expressed as the mean ± SD. ^***^p < 0.001

**Additional file 8:** **Figure S5. **circCUL2-overexpression NFs promote PDAC progression in vivo, related to Fig. 4. (A) Representative Bioluminescence images, lung and HE staining of lung tissue of mice 4 weeks after tail vein injection of luc-MiaPaCa-2 cells treated with conditioned medium as indicated (n = 8 per group). Scale bar, 100 μm. (B) Relative luminescence intensity in each group. (C) Histogram analysis of the metastatic nodules number in per lung. (D) lung metastasis rate of each group (Chi-square test). (E) Diagram of orthotopic xenograft model design. In brief, luc-PANC-1 cells or MiaPaCa-2 were co-injected with empty vector or circCUL2-transduced NFs. 3 days after injection, mice were treated with IL6 neutralizing antibodies (2mg/kg) everythree days. 30 days after implantation, original tumor and metastases were detected by in vivo imaging system. (F-G) Representative bioluminescence images and histogram analysis of luminescence intensity in each at day 30 are shown (n= 6). (H) Abdominal metastasis rate was calculated for indicated group(Chi-square test). (I) Representative images of orthotopic model in each group on which autopsy was performed. Red arrow indicated primary tumor; S, spleen; T, primary tumor; M, metastasis. (J) Timeline schematic for treatment of PDX mice. Arrows indicate different treatment time points. (K-L) qPCR and Western blot analysis of circCUL2 and IL6 expression in different PDX tumors.

**Additional file 9:** **Figure S6. **circCUL2 is a sponge of miR-203a-3p, related to Fig. 5.(A) qRT–PCR analysis of CUL2 mRNA enriched with biotin-labeled miR-203a-3p probes in NFs and CAFs.(B-C) qRT–PCR and western blot analysis of CUL2 in NF stransfected with miR-203a-3p inhibitor or in CAFs transfected with miR-203a-3p mimic. (D) The standard curves for copy number analysis of circCUL2 and miR-203a-3p were shown. (E) The average circCUL2 and miR-203a-3p copies per NFs and CAFs. Data are expressed as the mean ± SD. NS, no significant.

**Additional file 10:** **Figure S7. **miR-203a-3p is critical to maintain CAFs pro-tumor activity in vitro, related to Fig. 6.(A-B) Colony formation and transwell assays of MiaPaCa-2 cells treated with conditioned medium from miR-203a-3p-silencing NFs or miR-203a-3p-overexpression CAFs. Scale bar: 100 μm. Data are expressed as the mean ± SD. ^***^p < 0.001.

**Additional file 11:** **Figure S8. **circCUL2 promotes proliferation and migration via MyD88/NF-κB/IL6 axis, related to Fig. 7.(A-B) MiaPaCa-2 cells were treated with conditioned medium from NFs cotransfected circCUL2 overexpression plasmid with the miR-203a-3p mimic, or CAFs cotransfected circCUL2 siRNA and miR-203-3p inhibitor for 48 h. The proliferation and migration ability of MiaPaCa-2 were detected by colony formation and transwell assays. (C-D) MiaPaCa-2 cells were treated with conditioned medium from NFs cotransfected circCUL2 overexpression plasmid with MyD88 siRNA, or CAFs cotransfected circCUL2 siRNA and MyD88 overexpression plasmid for 48 h. The proliferation and migration ability of MiaPaCa-2 were detected by colony formation and transwell assays. Data are expressed as the mean ± SD. ^*^p < 0.05, ^**^p< 0.01 and ^***^p < 0.001.

**Additional file 12:** **Figure S9. **Clinical implication of circCUL2/miR-203a-3p/IL6 axis inPDAC, related to Fig. 8. (A) Correlation analysis of circCUL2 with miR-203a-3p in 161 PDAC patients. (B) Correlation analysis of circCUL2 with MyD88 in 161 PDAC patients. (C) Correlation analysis of circCUL2 with IL6 in 161 PDAC patients. (D) Correlation analysis of miR-203a-3p with IL6 in 161 PDAC patients.

## Data Availability

The datasets used in current study are available from the corresponding author on reasonable request.

## Abbreviations

PDAC: Pancreatic ductal adenocarcinoma
NFs: Normal fibroblasts
CAFs: Cancer-associated fibroblasts
iCAF: Inflammatory cancer-associated fibroblasts
myCAF: Myofibroblastic cancer-associated fibroblasts
circRNAs: Circular RNAs
LN: Lymph node
NAT: Normal adjacent tissues
OS: Overall survival
DFS: Disease-free survival
FISH: Fluorescence in situ hybridization
