# Supplementary material for: circCUL2 induces an inflammatory CAF phenotype in pancreatic ductal adenocarcinoma via the activation of the MyD88-dependent NF-κB signaling pathway
Source: J Exp Clin Cancer Res. 2022 Feb 21;41:71. doi: 10.1186/s13046-021-02237-6 (PMC8862589; doi:10.1186/s13046-021-02237-6)
Supplement: Supplementary file 1 — Additional file 1. [file 13046_2021_2237_MOESM1_ESM.docx]

**Table S1. Primers used in PCR.**

| **Primer Name** | **Sense (+)**  **Antisense (-)** | **Sequence** |
| --- | --- | --- |
| circCUL2 | **+** | 5’-AGAATACAGCAAGGGTGCAGA-3’ |
|  | **-** | 5’-AGACATTGTGCAAGTGTAGTGT-3’ |
| CUL2 | **+** | 5’-AAGCGGACCTTCAGTATGGC-3’ |
|  | **-** | 5’-GGATGGCCTGAAGTGGTTCA-3’ |
| miR-203a-3p-specific primer | **+** | 5’-GUGAAAUGUUUAGGACCACU-3’ |
| MyD88 | **+** | 5’-GGCTGCTCTCAACATGCGA-3’ |
|  | **-** | 5’-CTGTGTCCGCACGTTCAAGA-3’ |
| IL6 | **+** | 5’-ACTCACCTCTTCAGAACGAATTG-3’ |
|  | **-** | 5’-CCATCTTTGGAAGGTTCAGGTTG-3’ |
| CCL2 | **+** | 5’-CAGCCAGATGCAATCAATGCC-3’ |
|  | **-** | 5’-TGGAATCCTGAACCCACTTCT-3’ |
| CXCL1 | **+** | 5’-AGCTTGCCTCAATCCTGCAT-3’ |
|  | **-** | 5’-CCTCTGCAGCTGTGTCTCTC-3’ |
| THBS1 | **+** | 5’-AGACTCCGCATCGCAAAGG-3’ |
|  | **-** | 5’-TCACCACGTTGTTGTCAAGGG-3’ |
| MIF | **+** | 5’-GTGGTGTCCGAGAAGTCAGG-3’ |
|  | **-** | 5’-GGCACGTTGGTGTTTACGAT-3’ |
| VEGF | **+** | 5’-AGGGCAGAATCATCACGAAGT-3’ |
|  | **-** | 5’-AGGGTCTCGATTGGATGGCA-3’ |
| TNFα | **+** | 5’-CCTCTCTCTAATCAGCCCTCTG-3’ |
|  | **-** | 5’-GAGGACCTGGGAGTAGATGAG-3’ |
| IL1α | **+** | 5’-TGGTAGTAGCAACCAACGGGA-3’ |
|  | **-** | 5’-ACTTTGATTGAGGGCGTCATTC-3’ |
| ACTA2 | **+** | 5’-ACAATGAGCTTCGTGTTGCC-3’ |
|  | **-** | 5’-TGGCTGGGACATTGAAAGTC-3’ |
| Axin2 | **+** | 5’-TACACTCCTTATTGGGCGATCA-3’ |
|  | **-** | 5’-TTGGCTACTCGTAAAGTTTTGGT-3’ |
| GAPDH  (convergent) | **+** | 5’-GTCATCCCTGAGCTGAACGG-3’ |
|  | **-** | 5’-GTCAAAGGTGGAGGAGTGGG-3’ |
| GAPDH  (divergent) | **+** | 5’-CACCACACTGAATCTCCCCT-3’ |
|  | **-** | 5’-ATTTCCTTCCCGGTTGCAAC-3’ |
| circCUL2  (convergent) | **+** | 5’-ACTTGCACAATGTCTTTGAAACCA-3’ |
|  | **-** | 5’-ACGGTCATTCCATGTTGCTCT-3’ |
