# Supplementary material for: circCUL2 induces an inflammatory CAF phenotype in pancreatic ductal adenocarcinoma via the activation of the MyD88-dependent NF-κB signaling pathway
Source: J Exp Clin Cancer Res. 2022 Feb 21;41:71. doi: 10.1186/s13046-021-02237-6 (PMC8862589; doi:10.1186/s13046-021-02237-6)
Supplement: Supplementary file 2 — Additional file 2. [file 13046_2021_2237_MOESM2_ESM.docx]

**Table S2. Oligonucleotide sequences for this study.**

| **Name** | **Sequence** |
| --- | --- |
| **si-RNA** | |
| si-NC | - |
| si-circCUL2 #1 | ACUGCUUAUAUAGAUUUCATT |
| si-circCUL2 #2 | GCUUAUAUAGAUUUCAACATT |
| si-MyD88 | CCGGCAACUGGAGACACAATT |
| miR-NC |  |
| miR-203a-3p mimic | GUGAAAUGUUUAGGACCACUAG |
| NC-inhibitor | - |
| miR-203a-3p  inhibitor | CUAGUGGUCCUAAACAUUUCAC |
| **Probe for RNA pull down, FISH** | |
| circCUL2 probe | CAAGUGUAGUGUUGAAAUCUAUAUAAGCAGUCCAUA |
| miR-203a-3p probe | GUGAAAUGUUUAGGACCACUAG |
