# Supplementary figures and images for: circCUL2 induces an inflammatory CAF phenotype in pancreatic ductal adenocarcinoma via the activation of the MyD88-dependent NF-κB signaling pathway
Source: J Exp Clin Cancer Res. 2022 Feb 21;41:71. doi: 10.1186/s13046-021-02237-6 (PMC8862589; doi:10.1186/s13046-021-02237-6)

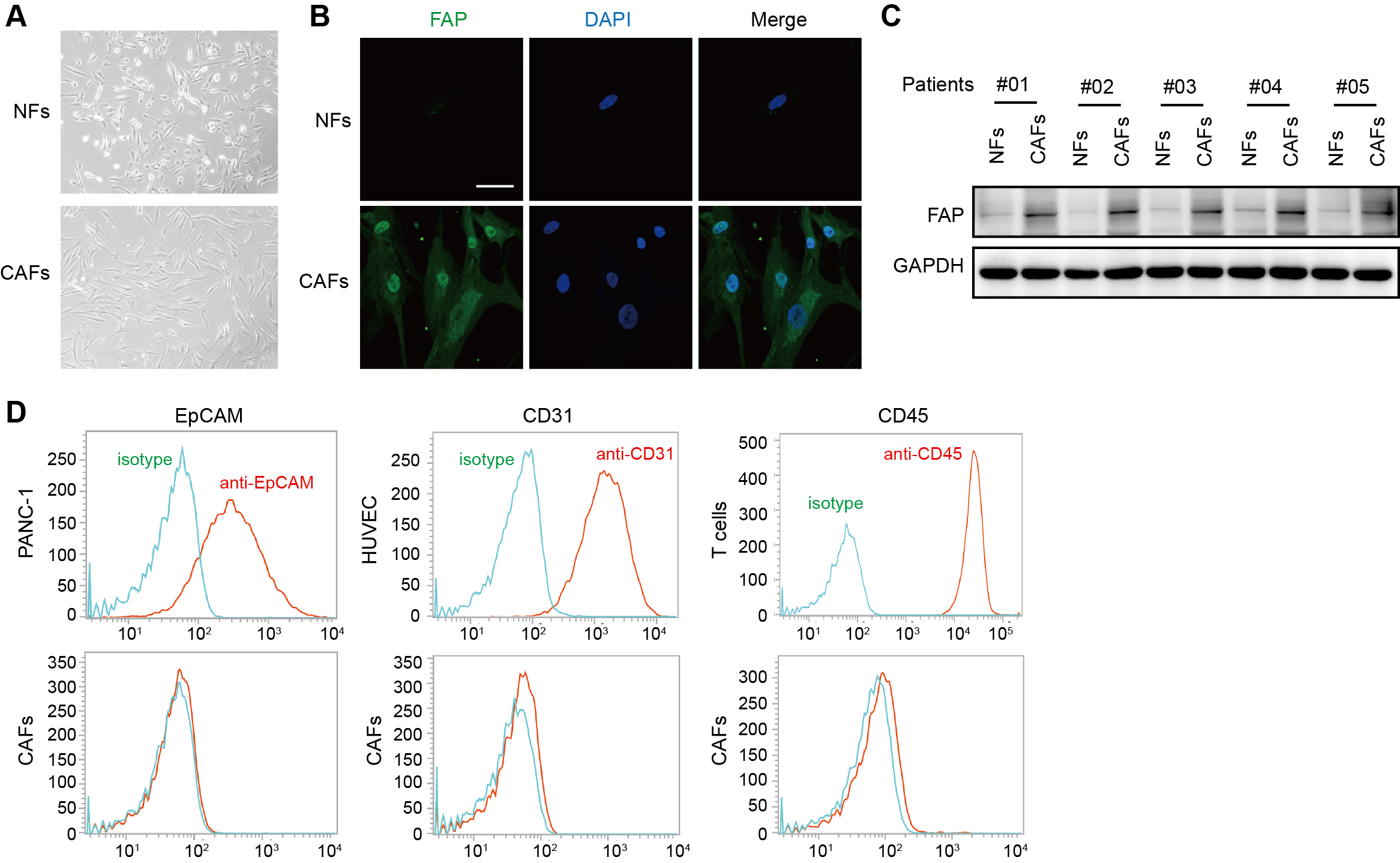

Supplement: Supplementary file 3 — Additional file 3: Figure S1. Isolation and identification of NFs and CAFs. (A) Morphology of NFs and CAFs isolated from clinical samples under light microscope. (B-C) Immunofluorescence and western blot analysis of FAP in NFs and CAFs isolated from clinical samples. Scale bar, 50 μm. (D) CAFs isolated from clinical samples were negative for EpCAM (epithelial marker), CD31 (endothelial marker) and CD45 (leukocyte marker), determined by flow cytometry. PANC-1 cancer cells, human umbilical vein endothelial cells (HUVEC) and human T lymphocytes were used as positive controls. Images for a representative sample were shown. [file 13046_2021_2237_MOESM3_ESM.tif]

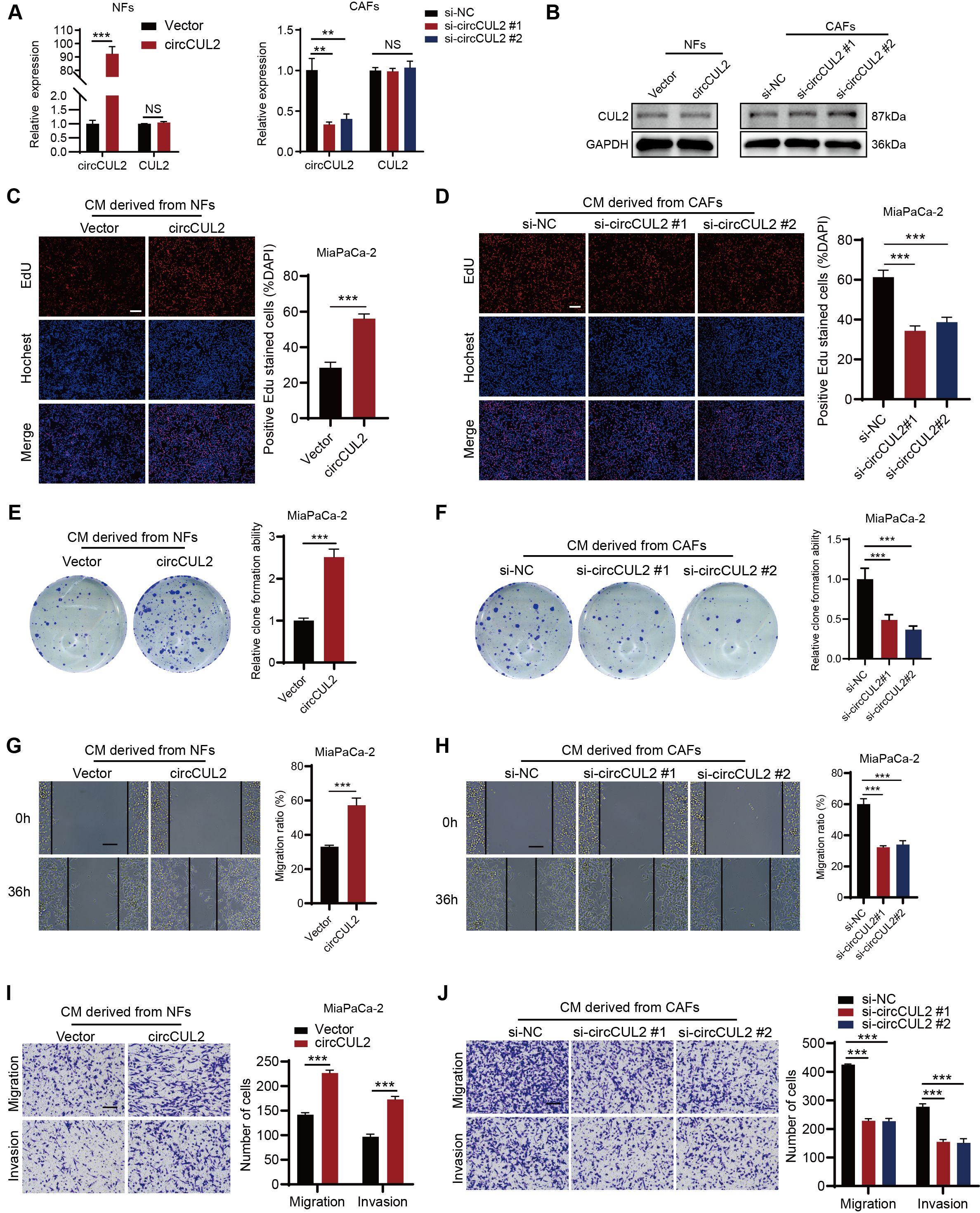

Supplement: Supplementary file 5 — Additional file 5: Figure S2. Specificity of circCU2L siRNA and overexpression vector, related to Fig. 2. (A) qRT–PCR analysis of circCUL2 and CUL2 expression following transfecting circCUL2 siRNA and overexpression vector inNFs and CAFs. (B) Western bolt analysis of CUL2 in NFs transfected with circCUL2 vector and in CAFs transfected with circCUL2 siRNA. (C-D) EdU assay of the proliferation of MiaPaCa-2 cells treated with conditioned medium from circCUL2-overexpression NFs or circCUL2-silencing CAFs. Scale bar: 100 μm. (E-F) Colony formation assays in MiaPaCa-2 cells treated with conditioned medium from circCUL2-overexpression NFs or circCUL2-silencing CAFs. (G-H) Scratch wound healing assays in MiaPaCa-2 cells treated with conditioned medium from circCUL2-overexpression NFs or circCUL2-silencing CAFs. Scale bar: 100 μm. (I-J) Transwell assays of migration and invasion of MiaPaCa-2 cells treated with conditioned medium from circCUL2-overexpression NFs or circCUL2-silencing CAFs. Scale bar: 100 μm. Data are expressed as the mean ± SD. **p <0.01 and ***p < 0.001. [file 13046_2021_2237_MOESM5_ESM.tif]

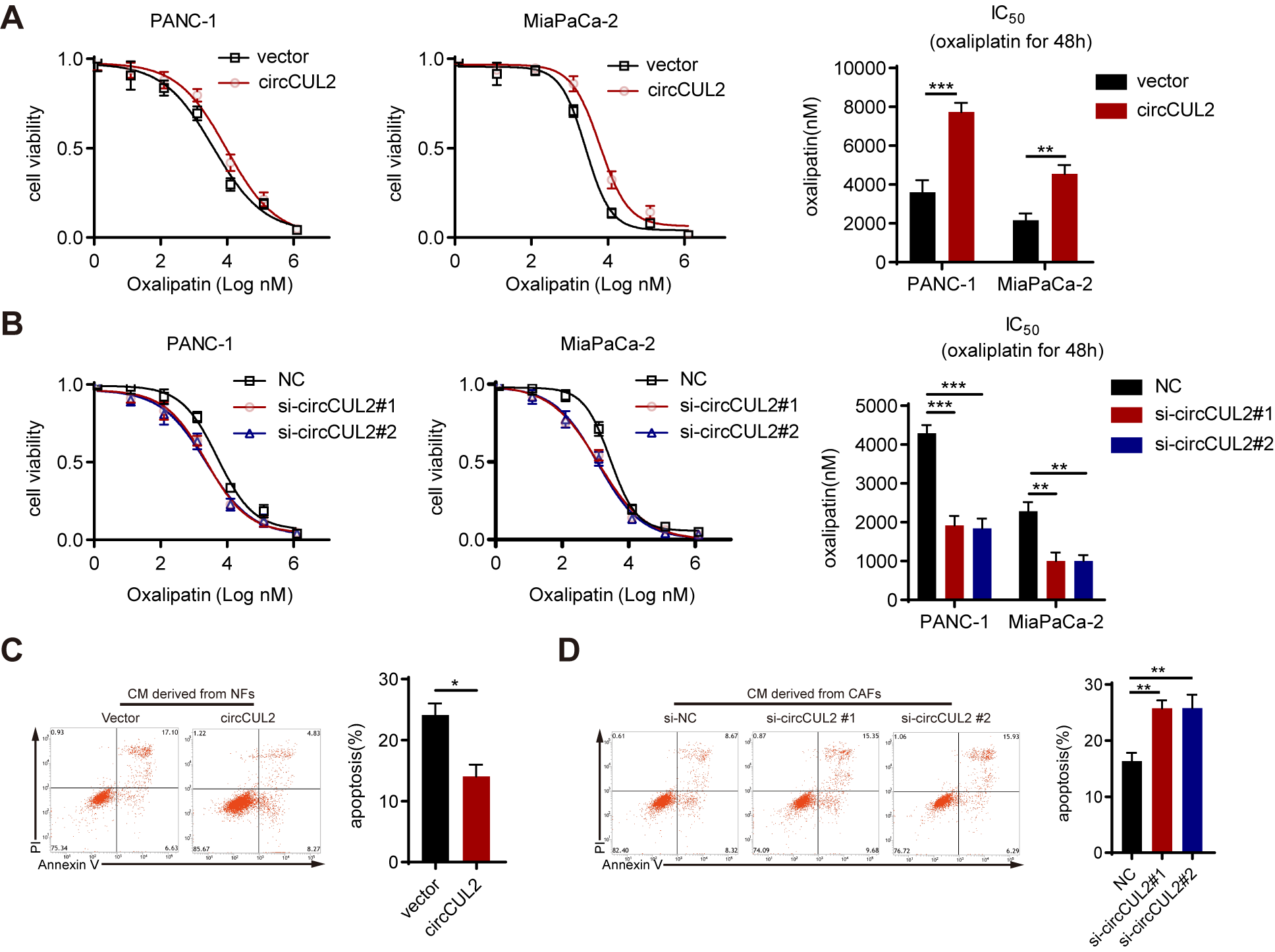

Supplement: Supplementary file 6 — Additional file 6: Figure S3. circCUL2 confers oxiaplatin resistance to PDAC cells. (A-B) Cell viability assay in PANC-1 and MiaPaCa-2 cells treated with conditioned medium from circCUL2-overexpression NFs or circCUL2-silencing CAFs. (C-D) Apoptosis assay in PANC-1 cells treated with conditioned medium from circCUL2-overexpression NFs or circCUL2-silencing CAFs. [file 13046_2021_2237_MOESM6_ESM.tif]

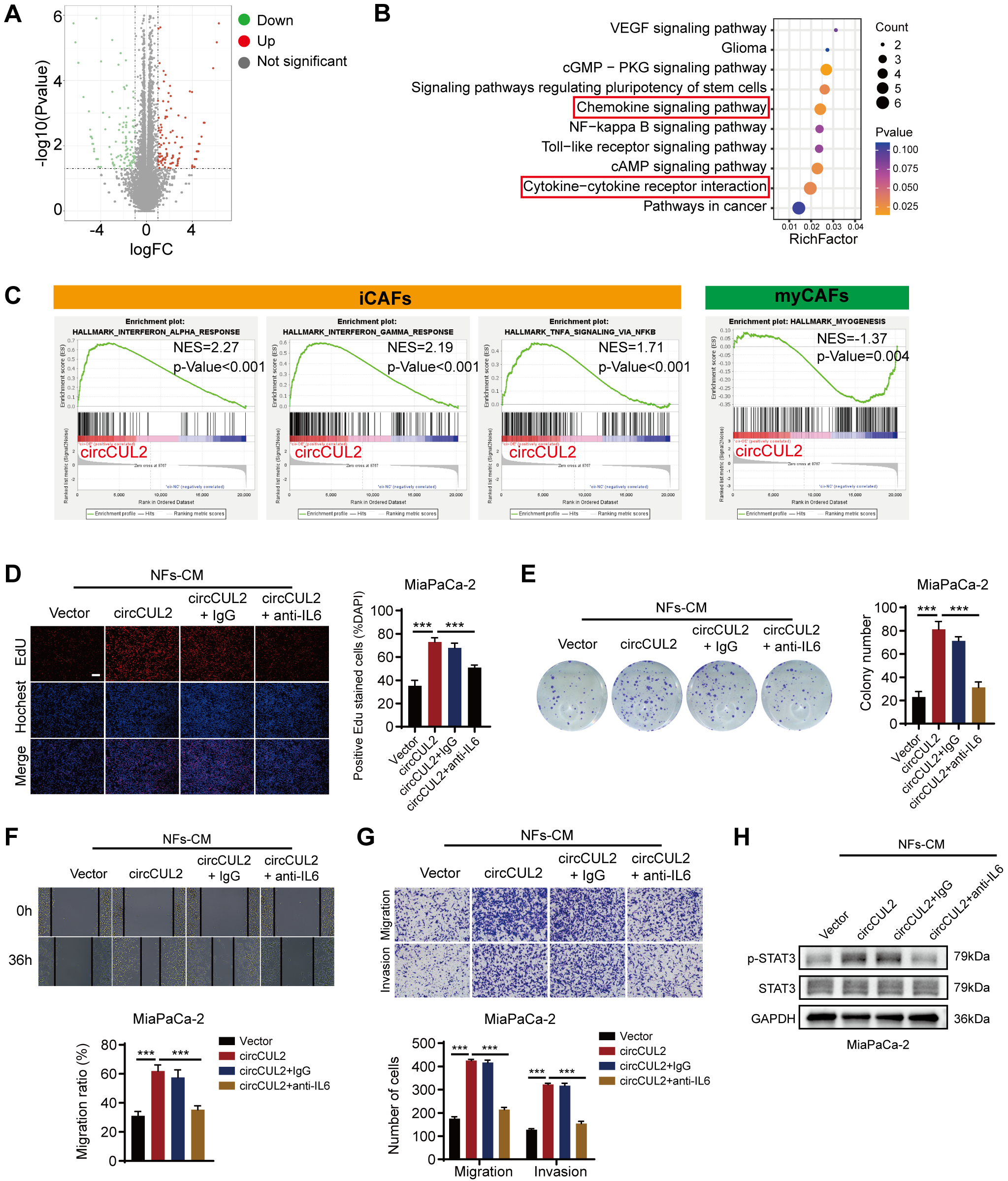

Supplement: Supplementary file 7 — Additional file 7: Figure S4. circCUL2 activates iCAF phenotype, related to Fig. 3. (A)Volcano plots of different expression genes in circCUL2-transducted NFs and empty vector-transduced NFs. different expression genes were selected by p <0.05 and fold-change >2. Gray dots indicated genes without significantly different expression, red dot indicated genes significantly up-regulated, and green indicated genes significantly down-regulated. (B) Enrichment of KEGG Pathway of different expression genes associated with cancer in circCUL2-transducted NFs. (C) GSEA plots for inflammatory CAF (iCAF) and myofibroblast-like CAF (myCAF) signatures in circCUL2 overexpression NFs, compared with control. (D-G) EdU assay (D), colony formation (E), Scratch wound healing assays (F) and transwell assays (G) of MiaPaCa-2 cells treated with conditioned medium from circCUL2-overexpression NFs or anti-IL6. Scale bar, 100μm. (H) western blot analysis of STAT3 and p-STAT3 in MiaPaCa-2 cells. Data are expressed as the mean ± SD. ***p < 0.001 [file 13046_2021_2237_MOESM7_ESM.tif]

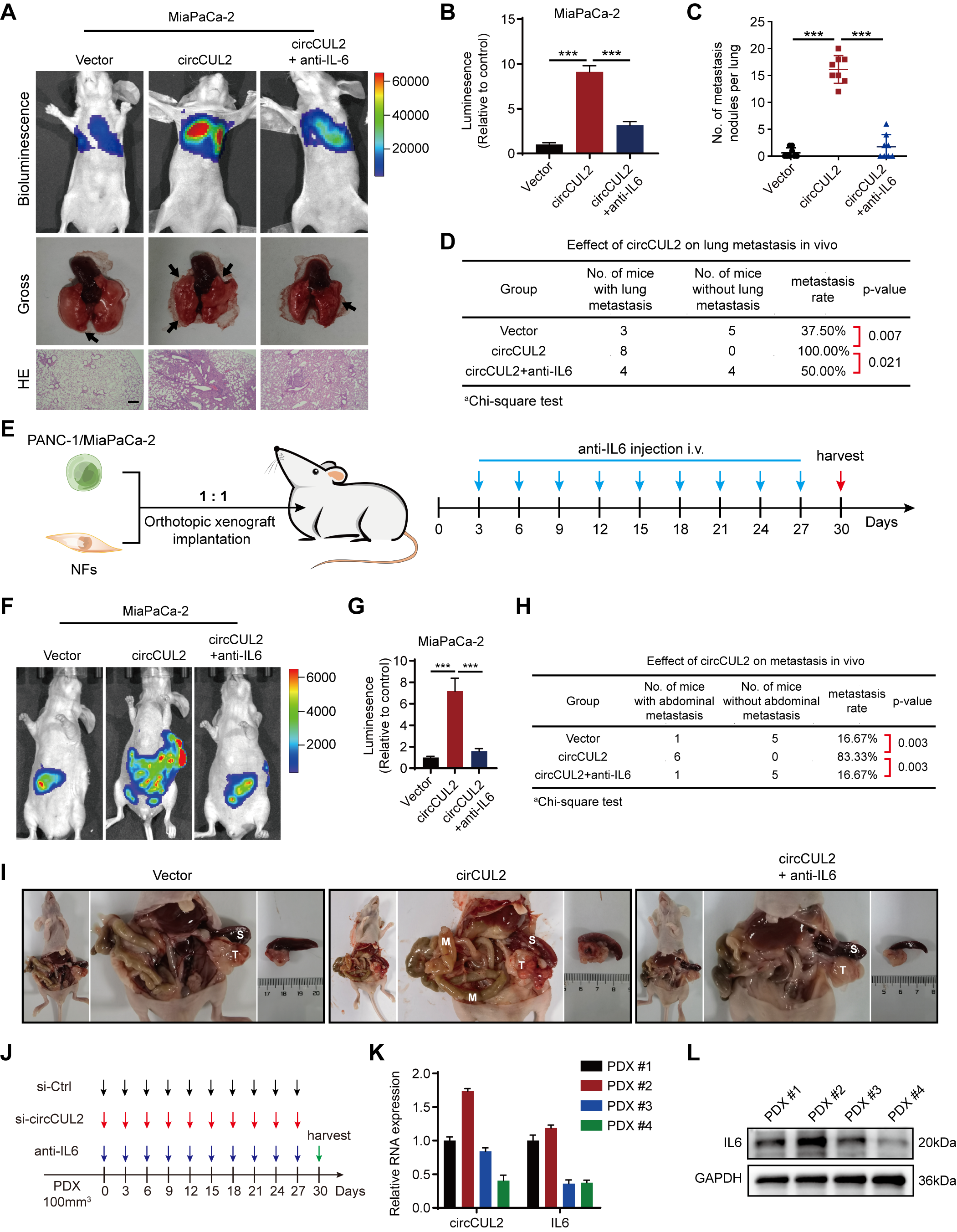

Supplement: Supplementary file 8 — Additional file 8: Figure S5. circCUL2-overexpression NFs promote PDAC progression in vivo, related to Fig. 4. (A) Representative Bioluminescence images, lung and HE staining of lung tissue of mice 4 weeks after tail vein injection of luc-MiaPaCa-2 cells treated with conditioned medium as indicated (n = 8 per group). Scale bar, 100 μm. (B) Relative luminescence intensity in each group. (C) Histogram analysis of the metastatic nodules number in per lung. (D) lung metastasis rate of each group (Chi-square test). (E) Diagram of orthotopic xenograft model design. In brief, luc-PANC-1 cells or MiaPaCa-2 were co-injected with empty vector or circCUL2-transduced NFs. 3 days after injection, mice were treated with IL6 neutralizing antibodies (2mg/kg) everythree days. 30 days after implantation, original tumor and metastases were detected by in vivo imaging system. (F-G) Representative bioluminescence images and histogram analysis of luminescence intensity in each at day 30 are shown (n= 6). (H) Abdominal metastasis rate was calculated for indicated group(Chi-square test). (I) Representative images of orthotopic model in each group on which autopsy was performed. Red arrow indicated primary tumor; S, spleen; T, primary tumor; M, metastasis. (J) Timeline schematic for treatment of PDX mice. Arrows indicate different treatment time points. (K-L) qPCR and Western blot analysis of circCUL2 and IL6 expression in different PDX tumors. [file 13046_2021_2237_MOESM8_ESM.tif]

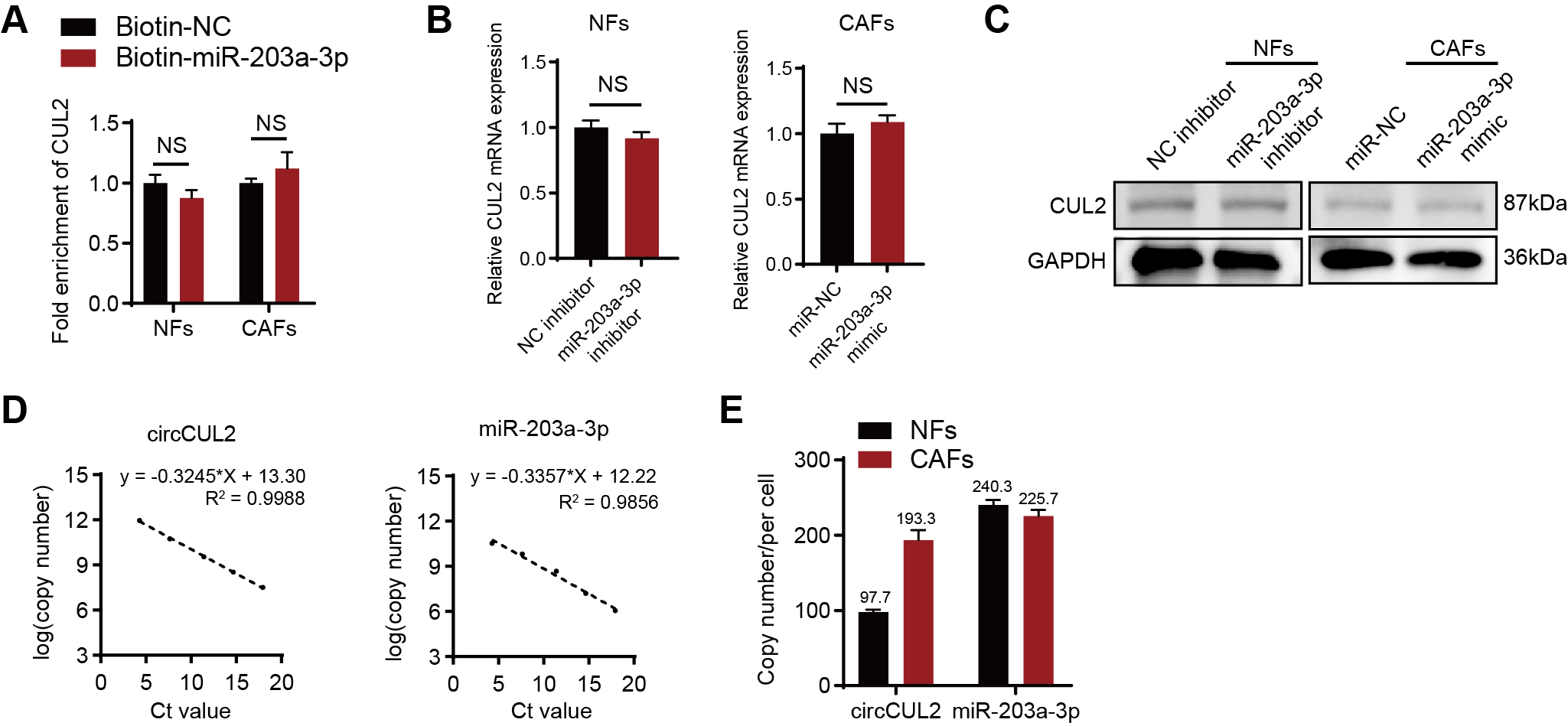

Supplement: Supplementary file 9 — Additional file 9: Figure S6. circCUL2 is a sponge of miR-203a-3p, related to Fig. 5.(A) qRT–PCR analysis of CUL2 mRNA enriched with biotin-labeled miR-203a-3p probes in NFs and CAFs.(B-C) qRT–PCR and western blot analysis of CUL2 in NF stransfected with miR-203a-3p inhibitor or in CAFs transfected with miR-203a-3p mimic. (D) The standard curves for copy number analysis of circCUL2 and miR-203a-3p were shown. (E) The average circCUL2 and miR-203a-3p copies per NFs and CAFs. Data are expressed as the mean ± SD. NS, no significant. [file 13046_2021_2237_MOESM9_ESM.tif]

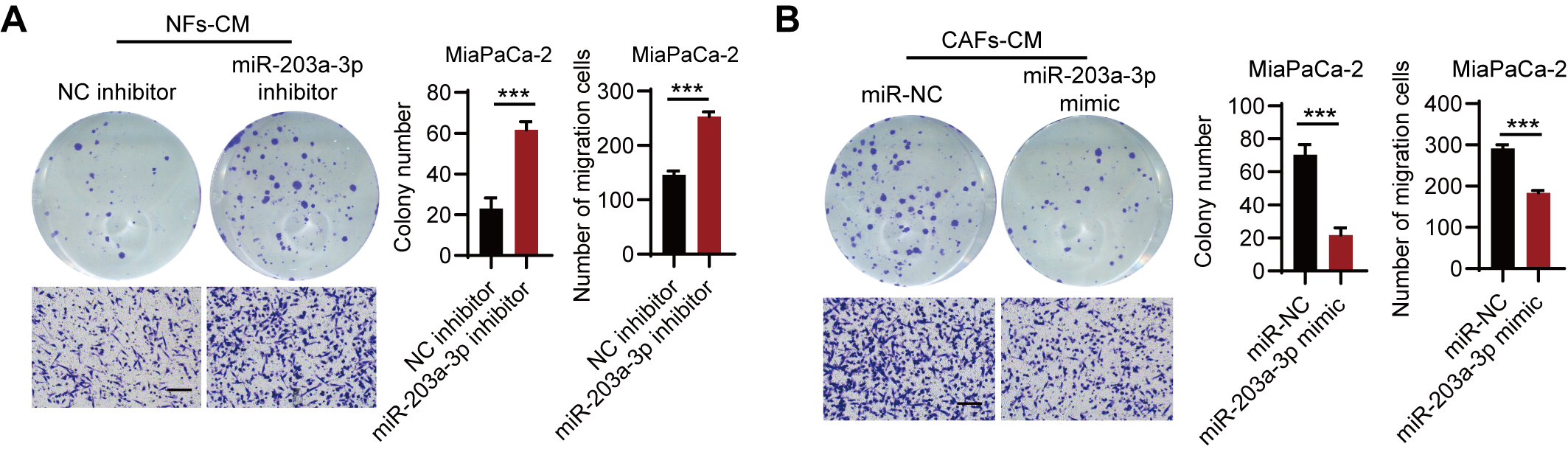

Supplement: Supplementary file 10 — Additional file 10: Figure S7. miR-203a-3p is critical to maintain CAFs pro-tumor activity in vitro, related to Fig. 6.(A-B) Colony formation and transwell assays of MiaPaCa-2 cells treated with conditioned medium from miR-203a-3p-silencing NFs or miR-203a-3p-overexpression CAFs. Scale bar: 100 μm. Data are expressed as the mean ± SD. ***p < 0.001. [file 13046_2021_2237_MOESM10_ESM.tif]

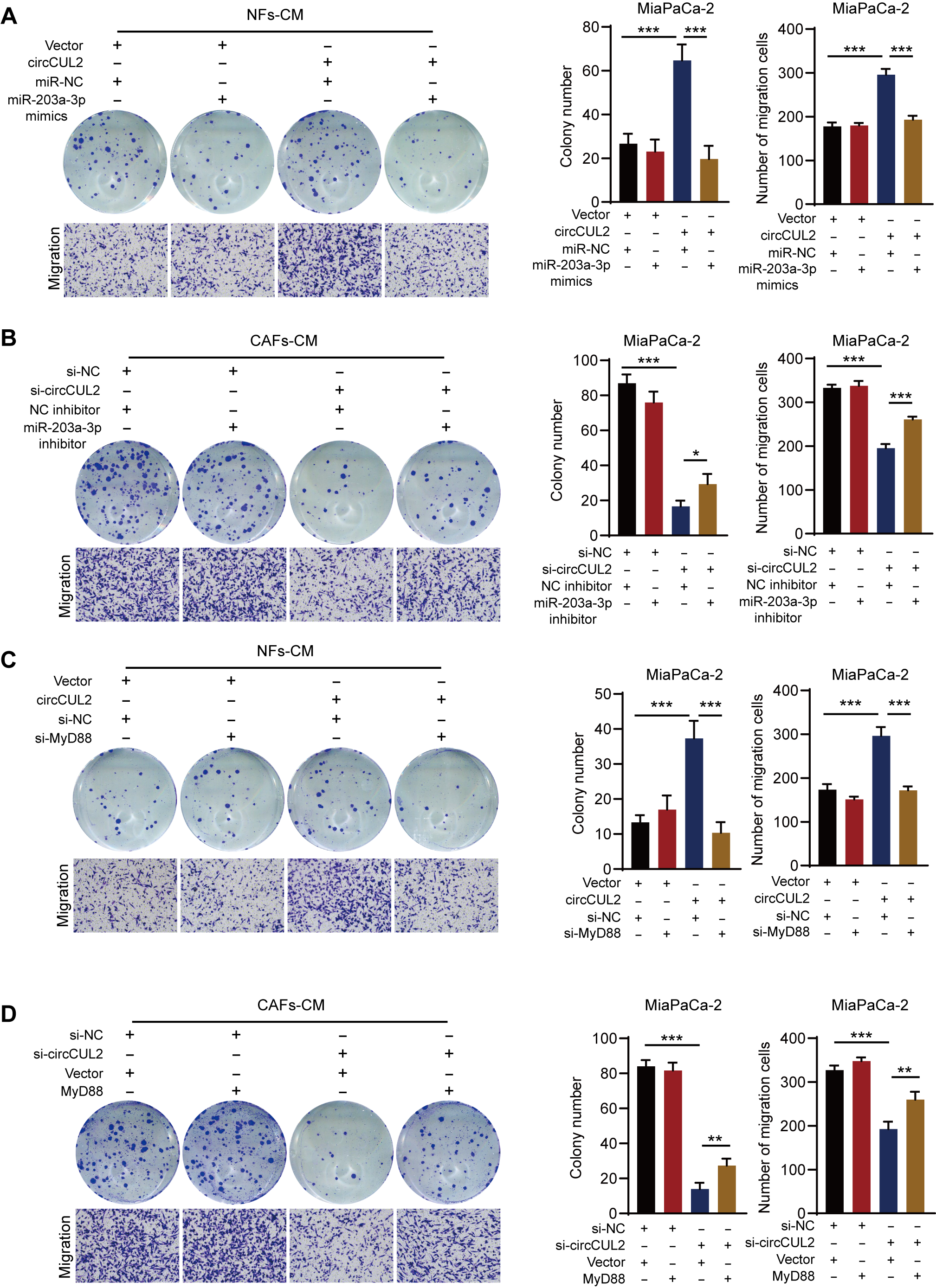

Supplement: Supplementary file 11 — Additional file 11: Figure S8. circCUL2 promotes proliferation and migration via MyD88/NF-κB/IL6 axis, related to Fig. 7.(A-B) MiaPaCa-2 cells were treated with conditioned medium from NFs cotransfected circCUL2 overexpression plasmid with the miR-203a-3p mimic, or CAFs cotransfected circCUL2 siRNA and miR-203-3p inhibitor for 48 h. The proliferation and migration ability of MiaPaCa-2 were detected by colony formation and transwell assays. (C-D) MiaPaCa-2 cells were treated with conditioned medium from NFs cotransfected circCUL2 overexpression plasmid with MyD88 siRNA, or CAFs cotransfected circCUL2 siRNA and MyD88 overexpression plasmid for 48 h. The proliferation and migration ability of MiaPaCa-2 were detected by colony formation and transwell assays. Data are expressed as the mean ± SD. *p < 0.05, **p< 0.01 and ***p < 0.001. [file 13046_2021_2237_MOESM11_ESM.tif]

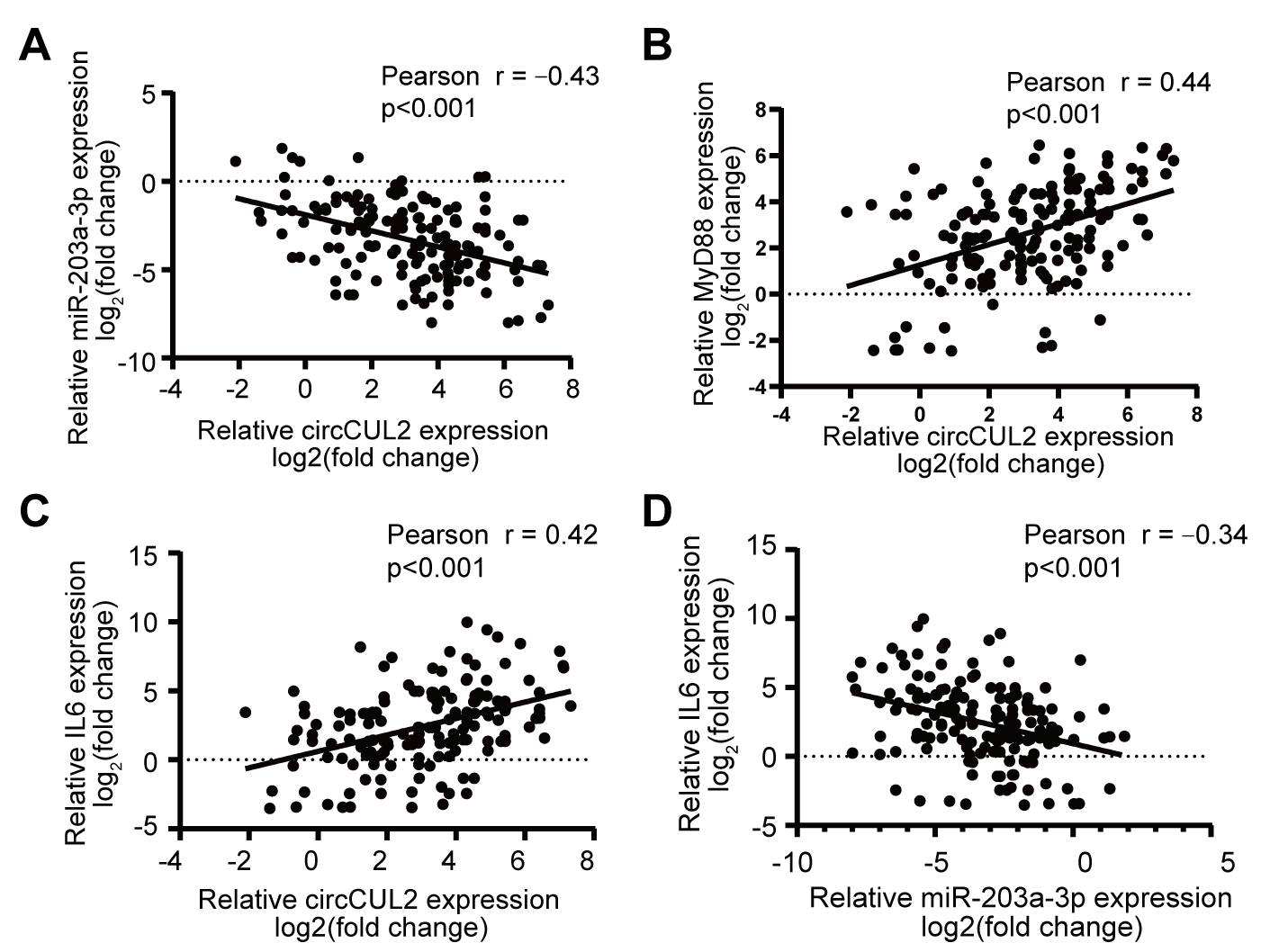

Supplement: Supplementary file 12 — Additional file 12: Figure S9. Clinical implication of circCUL2/miR-203a-3p/IL6 axis inPDAC, related to Fig. 8. (A) Correlation analysis of circCUL2 with miR-203a-3p in 161 PDAC patients. (B) Correlation analysis of circCUL2 with MyD88 in 161 PDAC patients. (C) Correlation analysis of circCUL2 with IL6 in 161 PDAC patients. (D) Correlation analysis of miR-203a-3p with IL6 in 161 PDAC patients. [file 13046_2021_2237_MOESM12_ESM.tif]
