# Supplementary material for: circCUL2 induces an inflammatory CAF phenotype in pancreatic ductal adenocarcinoma via the activation of the MyD88-dependent NF-κB signaling pathway
Source: J Exp Clin Cancer Res. 2022 Feb 21;41:71. doi: 10.1186/s13046-021-02237-6 (PMC8862589; doi:10.1186/s13046-021-02237-6)
Supplement: Supplementary file 4 — Additional file 4. [file 13046_2021_2237_MOESM4_ESM.docx]

**Table S3. qRT-PCR analysis of top 30 upregulated circRNAs in 1 pair of CAFs and NFs.**

| **circRNA ID** | **Fold change** | **p-Value** |
| --- | --- | --- |
| hsa_circ_0013730 | 2.15 | <0.001 |
| hsa_circ_0058644 | 9.13 | <0.001 |
| hsa_circ_0002557 | 6.21 | <0.001 |
| hsa_circ_0077441 | 1.96 | 0.011 |
| hsa_circ_0005838 | 1.14 | 0.186 |
| hsa_circ_0007919 | 1.43 | 0.426 |
| hsa_circ_0112170 | 1.16 | 0.575 |
| hsa_circ_0012269 | 1.72 | 0.025 |
| hsa_circ_0009684 | 1.34 | 0.296 |
| hsa_circ_0008886 | 2.13 | <0.001 |
| hsa_circ_0017451 | 1.34 | 0.341 |
| hsa_circ_0069739 | 1.54 | 0.016 |
| hsa_circ_0054503 | 1.35 | 0.361 |
| hsa_circ_0008062 | 7.94 | <0.001 |
| hsa_circ_0067974 | 1.52 | <0.001 |
| hsa_circ_0079614 | 1.30 | 0.463 |
| hsa_circ_0004539 | 0.61 | 0.100 |
| hsa_circ_0035642 | 1.13 | 0.788 |
| hsa_circ_0046840 | 1.24 | 0.046 |
| hsa_circ_0045220 | 1.35 | 0.435 |
| hsa_circ_0017704 | 1.06 | 0.679 |
| hsa_circ_0085392 | 1.67 | 0.099 |
| hsa_circ_0000234 | 10.89 | <0.001 |
| hsa_circ_0070982 | 1.66 | 0.054 |
| hsa_circ_0016070 | 1.65 | 0.007 |
| hsa_circ_0029803 | 1.50 | 0.100 |
| hsa_circ_0030573 | 5.66 | <0.001 |
| hsa_circ_0068088 | 1.51 | 0.214 |
| hsa_circ_0074678 | 1.75 | 0.050 |
